# Deep Learning‐Assisted Label‐Free Parallel Cell Sorting with Digital Microfluidics

**DOI:** 10.1002/advs.202408353

**Published:** 2024-11-05

**Authors:** Zongliang Guo, Fenggang Li, Hang Li, Menglei Zhao, Haobing Liu, Haopu Wang, Hanqi Hu, Rongxin Fu, Yao Lu, Siyi Hu, Huikai Xie, Hanbin Ma, Shuailong Zhang

**Affiliations:** ^1^ Beijing Advanced Innovation Center for Intelligent Robots and Systems, School of Mechatronical Engineering Beijing Institute of Technology Beijing 100081 China; ^2^ School of Medical Technology Beijing Institute of Technology Beijing 100081 China; ^3^ School of Integrated Circuits and Electronics Engineering Research Center of Integrated Acousto‐Opto‐Electronic Microsystems (Ministry of Education of China) Beijing Institute of Technology Beijing 100081 China; ^4^ CAS Key Laboratory of Bio‐Medical Diagnostics Suzhou Institute of Biomedical Engineering and Technology Chinese Academy of Sciences Suzhou 215163 China; ^5^ ACX Instruments Ltd, St John's Innovation Centre Cambridge CB40WS UK

**Keywords:** artificial intelligence, cell sorting, digital microfluidics, droplet, single cell research, label‐free

## Abstract

Sorting specific cells from heterogeneous samples is important for research and clinical applications. In this work, a novel label‐free cell sorting method is presented that integrates deep learning image recognition with microfluidic manipulation to differentiate cells based on morphology. Using an Active‐Matrix Digital Microfluidics (AM‐DMF) platform, the YOLOv8 object detection model ensures precise droplet classification, and the Safe Interval Path Planning algorithm manages multi‐target, collision‐free droplet path planning. Simulations and experiments revealed that detection model precision, concentration ratios, and sorting cycles significantly affect recovery rates and purity. With HeLa cells and polystyrene beads as samples, the method achieved 98.5% sorting precision, 96.49% purity, and an 80% recovery over three cycles. After a series of experimental validations, this method can also be used to sort HeLa cells from red blood cells, cancer cells from white blood cells (represented by HeLa and Jurkat cells), and differentiate white blood cell subtypes (represented by HL‐60 cells and Jurkat cells). Cells sorted using this method can be lysed directly on chip within their hosting droplets, ensuring minimal sample loss and suitability for downstream bioanalysis. This innovative AM‐DMF cell sorting technique holds significant potential to advance diagnostics, therapeutics, and fundamental research in cell biology.

## Graphical abstract

This paper presents a novel label‐free cell sorting method using Active‐Matrix Digital Microfluidics and YOLOv8 object detection model. The method demonstrates high purity and precision in sorting HeLa cells, red blood cells, and white blood cell subtypes, showcasing its potential for applications in cell sorting, omics research, single cell research, and drug development.

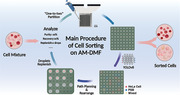

## 1 Introduction

Cell sorting, as a pivotal technique for isolating specific cell types or subpopulations from heterogeneous populations, plays a crucial role in advancing biomedical research, including cancer diagnostics,^[^ ^1^, ^2^, ^3^ ^]^ stem cell research,^[^ ^4^, ^5^ ^]^ and regenerative medicine.^[^ ^6^, ^7^ ^]^ For instance, the precise isolation of circulating tumor cells (CTCs) from liquid biopsy samples directly influences cytomics analysis in lesion tracing‐based cancer diagnosis.^[^ ^8^ ^]^ Additionally, in clinical applications, label‐free cell sorting and separation methods without the use of antibodies are important for low‐cost and rapid blood‐based diseases diagnosis.^[^ ^9^ ^]^ Hence, the development of effective cell‐sorting technique has profound implications for advancing associated research endeavors.^[^ ^10^ ^]^

In recent decades, fluorescence‐activated cell sorting (FACS) has emerged as a predominant technique for cell sorting. Established based on flow cytometry, FACS utilizes fluorescent labeling with distinct excitation wavelengths to identify and sort target cells.^[^ ^11^ ^]^ In combination with complementary methodologies such as surface acoustic wave manipulation, intracellular markers, and microchannel‐based microfluidics, FACS enables the sorting of various cell types, including MCF‐7 cells, endocrine cells, and K562 cells.^[^ ^12^, ^13^, ^14^, ^15^ ^]^ Additionally, magnet‐activated cell sorting (MACS) has also garnered significant attention recently. In MACS, cells are tagged with antibodies that are linked to magnetic beads, allowing them to be separated from unlabeled background cells by applying a magnetic field.^[^ ^16^ ^]^ This technique has been extensively utilized in research fields such as sperm selection, stem cell separation,^[^ ^17^ ^]^ and bacteria classification.^[^ ^18^ ^]^ However, both methods show certain limitations in sorting resolution and complexity. For example, FACS compromises the spatial resolution of images and necessitates extended processing time, often resulting in reduced cellular morphological details. On the other hand, MACS relies on the labeling of cells with magnetic beads, and thus cannot sort multiple cell types simultaneously. Moreover, both techniques involve cell labeling that might affect cellular activity and physiological states, potentially constraining downstream experiments for various biological applications, such as single‐cell proteomics.

To overcome these challenges, innovative cell sorting techniques, such as label‐free image‐based cell sorting (IBCS) has attracted a lot of research interest recently. This approach couples microfluidics and microarrays with optical imaging to improve sorting precision and efficiency.^[^ ^19^ ^]^ Cells are classified based on their morphological features through image processing.^[^ ^20^, ^21^, ^22^, ^23^, ^24^, ^25^, ^26^, ^27^ ^]^ Although current IBCS methods, such as microfluidic IBCS and microarray IBCS (as detailed in Table S1, Supporting Information), facilitate label‐free cell sorting, further improvements in automation, spatial resolution, and all‐in‐one cell manipulation are still in great need.

As a highly integrated and fully automated platform for biomedical analysis, digital microfluidics (DMF) has found extensive applications across various domains, including molecular diagnostics,^[^ ^28^, ^29^, ^30^, ^31^ ^]^ single‐cell‐omics,^[^ ^32^, ^33^, ^34^ ^]^ blood testing,^[^ ^35^, ^36^, ^37^, ^38^ ^]^ etc.^[^ ^39^, ^40^, ^41^, ^42^ ^]^ Traditional DMF devices utilize the electrowetting‐on‐dielectric (EWOD) effect to achieve precise manipulation of microliter‐sized droplets, facilitating tasks such as movement, splitting, and merging the droplets. Established on the principles of EWOD, researchers have integrated thin‐film transistor (TFT) technology to develop the active matrix (AM)‐DMF consisting of tens of thousands of tiny electrodes, which can be used to manipulate nL even pL droplets.^[^ ^43^, ^44^ ^]^ In recent years, the maturation and widespread adoption of artificial intelligence (AI) technology have led to the integration of microfluidics with computer vision AI models. This integration has advanced research in flow cytometry imaging,^[^ ^45^ ^]^ cell sorting,^[^ ^46^, ^47^ ^]^ cell viability assessment,^[^ ^48^, ^49^ ^]^ immunoassays,^[^ ^50^ ^]^ and various other fields.^[^ ^51^, ^53^ ^]^

**Figure 1:**
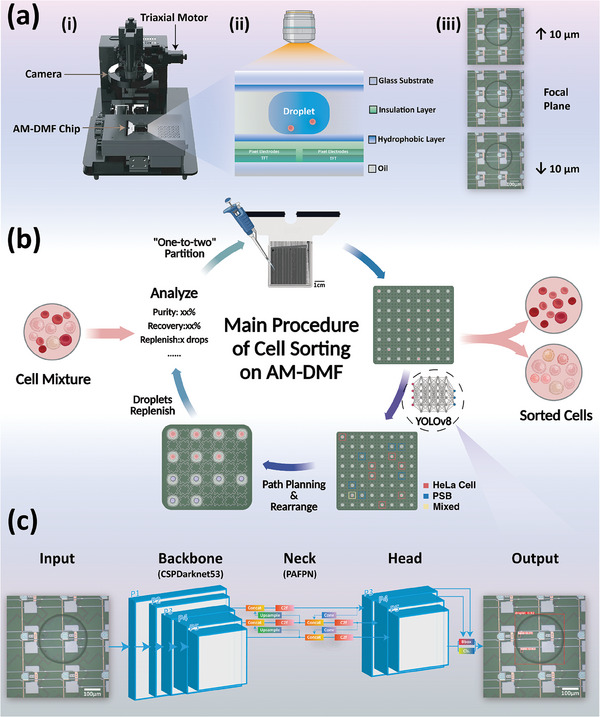
Illustration of cell sorting on AM‐DMF. (a) System setup. (i) AM‐DMF system. (ii) Cross‐section view of AM‐DMF chip. (iii) Images of the droplet captured at different heights. (b) Schematic workflow of cell sorting on AM‐DMF. (c) The structure and prediction workflow of YOLOv8 object detection model for cell identification.

Here, we present a novel label‐free cell sorting approach that integrates deep learning object detection with DMF manipulation to differentiate cells based on their morphological features. Utilizing an AM‐DMF platform (see **Figure** **1**a), we have established a novel YOLOv8 object detection model (prediction workflow and network structure shown in Figure 1c, with details in Figure S1, Supporting Information) for accurate classification of cells within nL droplets, and a Safe Interval Path Planning algorithm (SIPP) to control the movement of these droplets and achieve collision‐free cell separation on chip. The sorting process mainly consists of five steps (see Figure 1b), and its efficiency can be continuously improved by introducing fresh droplets into the system (as detailed in Movie S1, Supporting Information). This method enables rapid, all‐in‐one, label‐free cell sorting, achieving sorting purity greater than 96% and a recovery rate exceeding 80% for HeLa cells and polystyrene beads (PSBs) mixtures. Furthermore, we applied this method to sort mixtures of red blood cells（RBCs) and HeLa cells, HeLa cells and Jurkat cells, and HL‐60 cells and Jurkat cells, demonstrating its potential to work with different cell samples.

## 2 Results and Discussion

### 2.1 Evaluation of Object Detection Models using Simulation

The two most important parameters for cell sorting methods are rates of recovery and purity.^[^ ^54^ ^]^ Additionally, for image‐based approaches, the primary factors influencing the sorting performance are the precision of the object detection model and the concentration ratio of target cells to background cells. To evaluate these parameters, we performed a series of experimental simulations on model samples to assess the influence of detection precision and concentration ratio on recovery and purity.

First, the distribution of PSBs in droplets were simulated. As the electrode of the AM‐DMF chip is 250 µm in width and with a depth of 50 µm, the total volume of each droplet in the 64‐droplet array is equivalent to 0.2 µL. Typically, the distribution of cells in liquid droplets follows a Poisson distribution, with λ representing the average number of cells per droplet.^[^ ^55^ ^]^ In our mathematical modeling (as detailed in the Supporting information), we defined a loading concentration of 3.2 × 10^5^ cells per mL. Accordingly, 64 cells were distributed within the droplets, resulting in an average of one cell per droplet. For a 1:1 HeLa cells to PSBs mixture, the number of HeLa cells and PSBs in each droplet follows an independent and identically distributed (i.i.d) Poisson distribution with λ  =  1. For a ratio of HeLa cells to PSBs at 1: r, the distribution of PSBs follows a Poisson distribution with λ  =  1/*r*.

**Figure 2:**
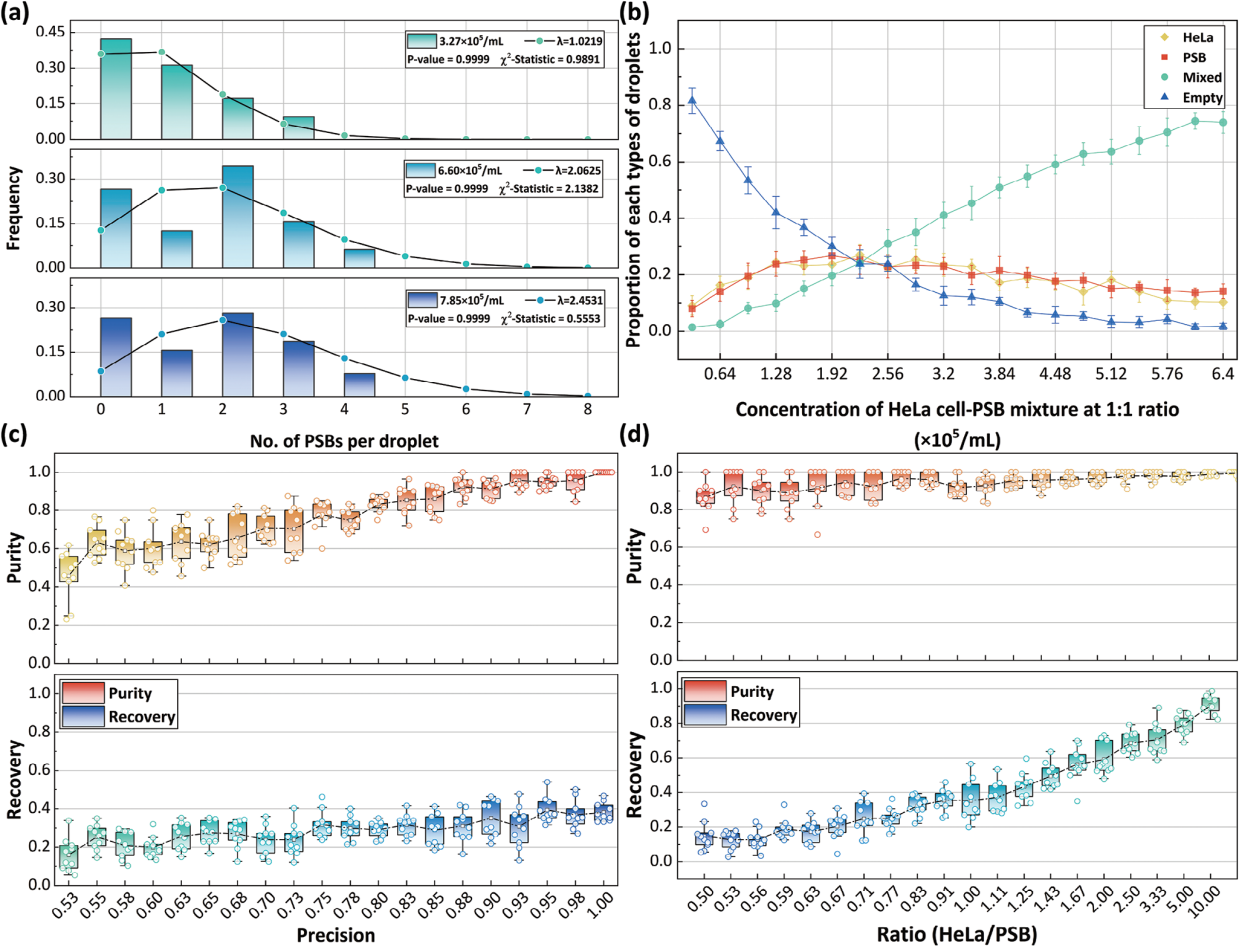
Impact of precision and ratio of target to background cells on the purity and recovery rate. (a) The distribution of PSBs in a droplet array for PSB samples at different concentrations. Insets show the *p*‐values of K‐S test,^[^ ^57^ ^]^ and λ values for the Poisson distribution curves. (b) The proportion of four distinct types of droplets across varying mixture concentrations for a 1:1 HeLa cells‐PSBs mixture. (c, d) Influence of object detection model precision and the ratio of HeLa cells to PSBs on sorting purity and recovery rate of HeLa cells.

To validate the suitability of the derived Poisson distribution for modeling the droplets containing PSBs, we conducted on‐chip experiments. PSBs at different concentrations were loaded onto the chip, split into single droplet arrays, and analyzed using the Chi‐square test.^[^ ^56^ ^]^ As illustrated in **Figure** **2**a, the distribution of PSB quantities within droplets follows a Poisson distribution. The values of λ were determined as 1.219, 2.0625, and 2.4531 for different PSB concentrations, respectively. This result suggests that a rise in PSB concentration correlates with an increased average number of PSBs per droplet. All *p*‐values were significantly greater than 0.05, validating that the distribution of droplets obeys the Poisson distribution.

Subsequently, simulations were performed to analyze the impact of sample concentration on the distribution of different types of droplets throughout the cell sorting process. For λ values ranging from 0.1 to 2 in increments of 0.1, the mixture concentration was adjusted from 0.32 × 10^5^ to 6.4 × 10^5^ cells per mL with an increment of 0.32 × 10^5^ cells per mL. The proportions of distinct droplet types, including droplets containing only HeLa cells, only PSBs, a mix of both, and empty droplets (those without cells and PSBs), were quantified and plotted in Figure 2b. The proportion of droplets with a mix of HeLa cells and PSBs increases sharply with increasing concentration. Since the efficacy of cell sorting highly depends on the number of droplets containing only the target cells, a higher percentage facilitates the isolation of target cells with a single sorting process. The increased proportion of mixture droplets might result in large number of target cells difficult to be sorted, requiring more sorting cycles and leading to a lower recovery rate. Conversely, at low concentrations, most droplets consist of either only HeLa cells, PSBs, or no objects at all, facilitating one round of sorting to capture most target cells. This observation indicates that this approach is more suitable for samples with low cell concentrations.

Next, we examined the impact of detection model precision on the recovery rate and purity of target cells for a 1:1 HeLa cells‐PSBs mixture. For cells within each droplet, the object detection model has a misclassification probability of 1‐precision (one minus the probability value of precision). We systematically varied the precision from 0.525 to 1.000 with a step size of 0.025, conducting 20 sets of simulation, each repeated 10 times. The resulting recovery and purity values are presented in Figure 2c. Generally, the precision of object detection model markedly influences the sorting purity while slightly affects the recovery. It shows that higher precision is associated with improved sorting purity, whereas lower precision might result in more misclassified background cells, reducing sorting purity.

Further, we set the precision to 0.9 and varied the ratio of HeLa cells to PSBs from 5 to 0.5 for 20 sets of simulations, each repeated ten times. The results exhibit that a higher percentage of target cells correlates with a higher recovery rate and purity, as shown in Figure 2d. Moreover, the ratio of HeLa cells to PSBs more significantly impacts the recovery rate than the sorting purity. As the modeling demonstrated, the ratio of target cells to background directly influences the droplet content distribution. Higher ratios yield more droplets with only HeLa cells or PSBs, facilitating the sorting of HeLa cells and consequently enhancing the recovery rate. These findings suggest that a high‐precision object detection model is essential for effective cell sorting. It is also recommended to preprocess mixture samples to remove background cells, such as preliminary enrichment of blood samples for sorting CTCs, to improve sorting purity.

### 2.2 Comparison and Optimization of Object Detection Models

**Figure 3:**
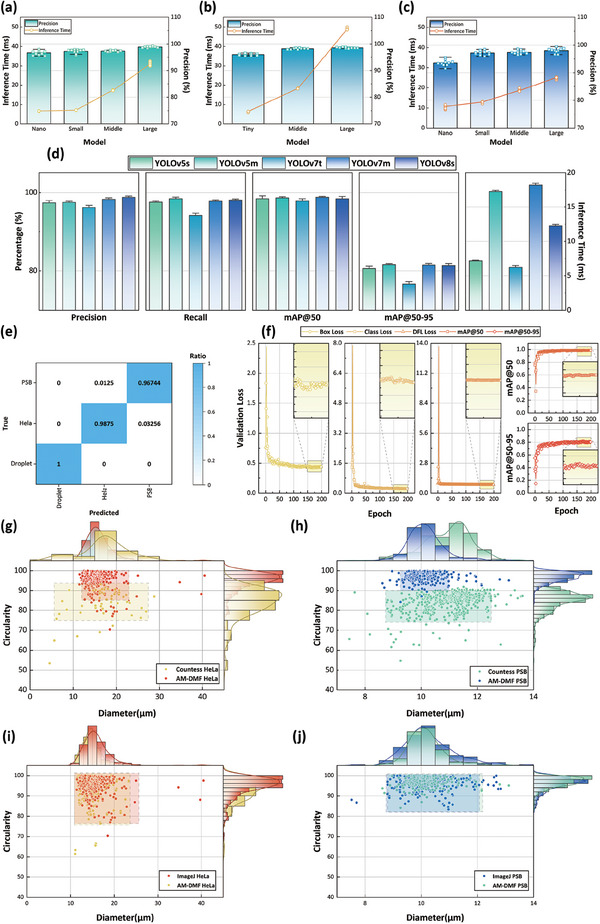
Model training evaluation. (a–c) Inference time and precision for the different sizes of the YOLOv5, ‐v7, and ‐v8 model, respectively. (d) Performance of different versions of the YOLO model on metrics of precision, recall, mAP@50, mAP@50‐95, and inference time. (e) Confusion matrix of YOLOv8s of the training on the HeLa‐PSB dataset. The average precision of the three types of objects is 0.985. (f) Trends in each metric when the training epoch is 200, and details of training for the next 50 epochs. (g–j) Marginal histogram of circularity and diameter of the sorted HeLa cells and PSBs obtained using the deep learning image recognition‐based AM‐DMF, Countess III assay, and ImageJ, respectively.

To establish and optimize a high‐precision object detection model, we compared three YOLO‐based algorithms, including YOLOv8, ‐v7,^[^ ^58^ ^]^ and ‐v5 (https://github.com/ultralytics/yolov5). We performed ten repetitions of training for each model with different parameter sizes (as detailed in Table S2, Supporting Information) using the same HeLa‐PSBs dataset. **Figure** **3**a–c shows the precision and inference time of YOLOv5, v7, and v8 models with different parameter sizes, respectively. Among the different sizes of YOLOv8, the “small” model was identified as the most efficient, providing high precision with relatively low runtime, which is crucial for real‐time, high‐purity cell sorting. Similarly, the YOLOv7‐tiny, YOLOv7‐middle, YOLOv5‐small, and YOLOv5‐middle performed better than their counterparts of different sizes. We further compared the precision, inference time, and other relevant metrics across these models, as shown in Figure 3d. The results demonstrate that YOLOv8 consistently outperforms other models, achieving an average precision of 98.5% and an average runtime of 12 ms per image. These metrics highlight YOLOv8's effectiveness in achieving high precision while maintaining low computational cost.

In‐depth analysis of the training dynamics for YOLOv8 is depicted in Figure 3e through a confusion matrix. This model achieves nearly perfect precision across all three categories present in the HeLa‐PSB dataset: droplets, HeLa cells, and PSBs. The training trajectory over 200 epochs, displayed in Figure 3f, shows a gradual reduction in the model's loss functions and a steady increase in both mAP@50 and mAP@50‐95 metrics. However, a detailed examination reveals a saturation in precision during the final 50 iterations, suggesting that extending the training beyond 200 epochs may not yield significant improvements. Additional details on the training and extended parameters are available in Figures S2 and S6 (Supporting Information).

### 2.3 On‐Chip Cell Sorting using YOLOv8

To further evaluate the performance of YOLOv8‐based AM‐DMF cell sorting, we performed additional experiments, aiming to compare their capabilities in morphology recognition and cell identification with those of the conventional Countess III cell counter.

Using the 399 images in the training dataset, we employed the YOLOv8 model and Countess III cell counter separately for object detection and extracting data on the bounding boxes (Bboxes). The marginal histograms in Figure 3g,h depict the diameter and circularity distributions of the HeLa cells and PSBs, respectively, using the two approaches. The distribution of HeLa cells with AM‐DMF predominantly fell within a diameter range of 12–23 µm and circularity between 85% and 100%. In contrast, the Countess III results for HeLa cells were primarily distributed between 6 and 27 µm in diameter, with circularity ranging from 75% to 90%. Similarly, PSBs were predominantly distributed within the 90%–100% circularity range and 9.3–11 µm in diameter using AM‐DMF, and within the 75%–90% circularity range and 8.7–12.5 µm in diameter using Countess III. This demonstrates that a tighter clustering of object diameter and circularity was observed with our approach, confirming its superior performance in depicting the true morphological features of HeLa cells and PSBs. In general, PSBs, as artificially engineered microbeads, exhibit a more consistent and tightly distributed diameter (predominantly 9–12.5 µm) and circularity (mainly >90%), as compared to HeLa cells, which inherently slightly vary in morphology, facilitating an effective sorting of the two types of objects. In addition, we employed ImageJ to measure the shape parameters of 480 PSB and 422 HeLa cells as a standard reference for cell shape measurement. The results were compared with those obtained using the AI detection model on AM‐DMF, as shown in Figure 3i,j. The strong alignment between the results from our method and the standard ImageJ measurements further validates the high accuracy of our AI cell detection model.

In conclusion, the cell detection outcomes for HeLa cells and PSBs on AM‐DMF prove significantly more precise and aligned with actual conditions compared to those generated by the Countess III cell counter. Thus, our proposed deep learning image recognition‐based AM‐DMF cell sorting approach has demonstrated the capability to achieve high‐precision detection and classification of target cells.

### 2.4 Iterative Cycles for Cell Sorting

**Figure 4:**
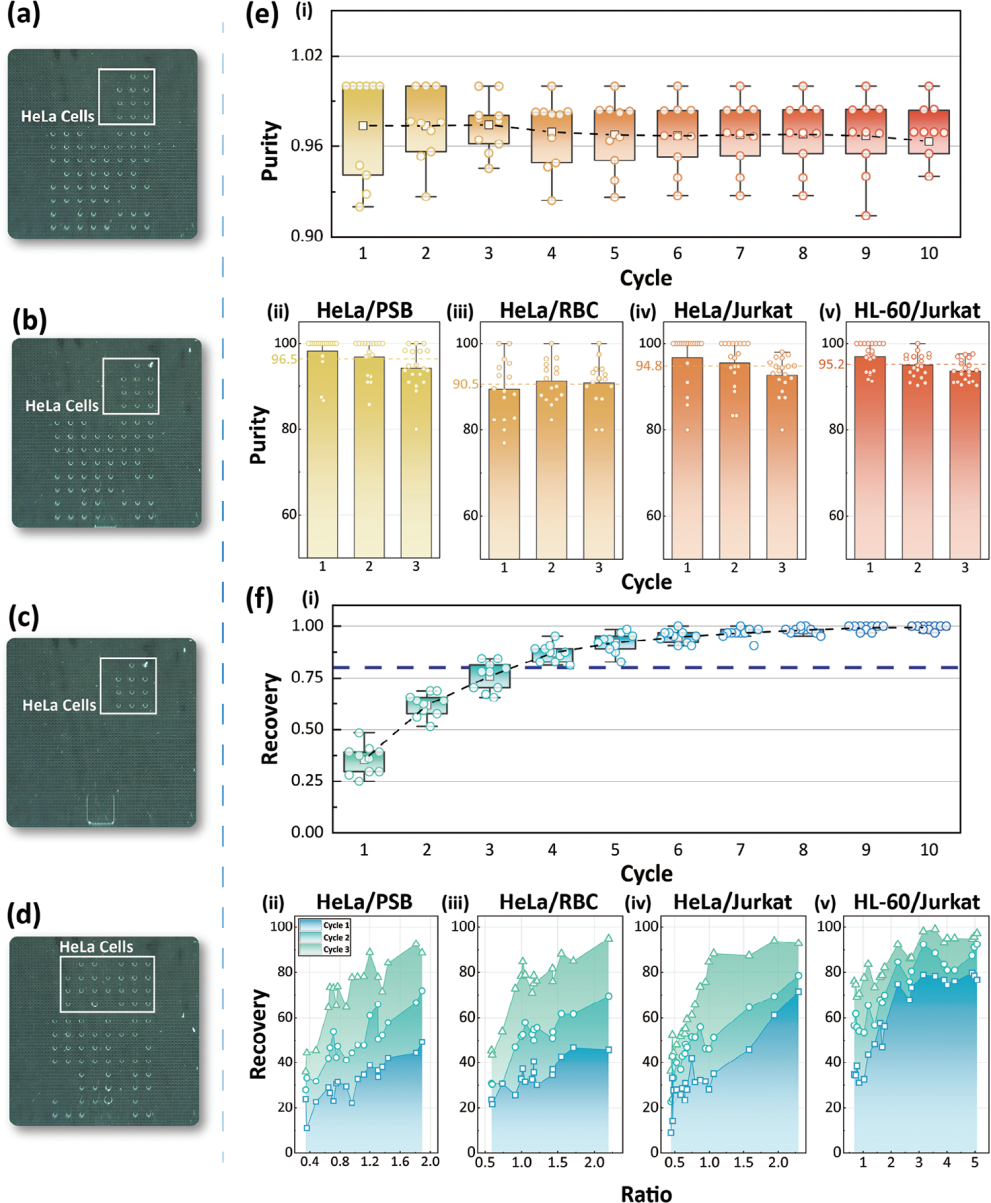
Influence of the number of sorting cycles on cell sorting results. (a–d) Images showing the progression of the cell sorting cycles, captured from Movie S1 (Supporting Information). (e). (i–iv) Simulation and experiment results display the sorting purity versus the number of cycles for HeLa‐PSB, HeLa‐RBC, HeLa‐Jurkat, and HL‐60‐Jurkat sorting, respectively. (f). (i–iv) Simulation and experiment results display the trend of sorting recovery rate with the number of cycles and ratios in simulation for HeLa‐PSB, HeLa‐RBC, HeLa‐Jurkat, and HL‐60‐Jurkat sorting, respectively.

Effective cell sorting can be achieved through iterative cycles. By periodically introducing fresh droplets, the cycles of cell sorting operations have been established for the proposed DMF approach (see **Figure** **4**a–d, Movie S2, Supporting Information). Since the injected empty droplets do not contain HeLa cells and PSBs, they function as diluents, thereby reducing the value of λ in the Poisson distribution as well as the number of HeLa cells or PSBs in the remained droplets. The following experiment was conducted to assess the change of recovery and purity trend. We set the ratio to 1 and the precision to 0.985, representing the true precision of the YOLOv8s object detection model trained on the HeLa‐PSB dataset. After replenishing the droplets, we recalculated the λ value as the average value of the remaining targets in the droplets. We iterated the cell sorting process 1–10 times and repeated each experiment 10 times to record recovery and purity. The experimental results are visualized in Figure 4e (i) and Figure 4h (i).

As the number of cycles increases, the purity remains steady while the recovery rate significantly improves. The recovery rate is defined as the ratio of collected HeLa cells to all HeLa cells in the sample. By introducing replenished droplets in cycles, the occurrence of mixed droplets decreases, resulting in more HeLa cells being sorted and, hence, an improved recovery rate. Purity is defined as the ratio of collected HeLa cells to all collected objects. Since each cell sorting process is independent, both the number of sorted HeLa cells and the total number of collected objects increase with cycles, leading to no significant change in purity. Overall, three to four iterations are sufficient to achieve a recovery rate of over 80%, which is relatively high. However, the time required also increases with the number of cycles. The optimal number of cycles should be determined based on specific application scenarios. Typically, for the sorting of rare cells, a higher number of cycles would be preferred to minimize the loss of target cells.

Furthermore, 19 sets of on‐chip cell sorting experiments were performed with different HeLa cell to PSBs ratios and varying numbers of cycles. The resulting recovery rates and purities are shown in Figure 4e (ii) and Figure 4f (ii), and detailed in Table S5 (Supporting Information). The recovery rate substantially increases with the number of cycles and enhances with an increased proportion of target cells, consistent with the above simulation results. In contrast, purity remains stable over cycles with a slight decreasing trend. This decrease can be attributed to the uncertainty in the position of the PSB within the droplet, potentially causing recognition errors, especially when the PSB is at the edge of the droplet, leading to an accumulation of errors as the sorting cycle proceeds. Additionally, the recovery rates obtained from the on‐chip experiments were slightly lower than the simulation results. This difference might be caused by various factors during the sorting process, such as insufficient replenishment of droplet volume to generate 8 × 8 droplet arrays and incidental loss of droplet manipulation by the AM‐DMF chip.

### 2.5 Biological Applications

To apply our proposed cell sorting method in biological studies, we tested the sorting of cancer cells from leukocytes, specifically sorting HeLa cells from Jurkat cells (an immortalized cell line of human T lymphocytes), and HL‐60 cells (a human promyelocytic leukemia cell line derived from neutrophils) from Jurkat cells, with both HeLa and HL‐60 cells present in small quantities. Details regarding the dataset construction and model training can be found in the “*Object Detection Model for Cell Classification*” of the Experimental Section. As shown in supplementary Figures S4–S9 (Supporting Information), the object detection models perform well in recognizing and classifying a variety of rare cells, with all precision rates exceeding 95%.

Using the established models, we performed on‐chip cell sorting at various stages and with three different ratios of HeLa cells to human RBCs, HeLa cells to Jurkat cells, and HL‐60 cells to Jurkat cells. The sorting results (see Figure 4e(iii–v), 4f(iii–v) and Tables S6–S8, Supporting Information) align with the findings from the HeLa‐PSB sorting analysis, although there was a slight decrease in sorting purity due to the lower precision of the object detection model for cell recognition. Nevertheless, the sorting purity remained above 90%. These results demonstrate the effectiveness of our strategy in isolating various rare mammalian cells, including subtypes of blood cells from leukocytes. This indicates the potential of our DMF approach for complex clinical applications, such as circulating tumor cell screening.

In addition, to improve the sorting performance for rare cells, we employed a pre‐sorting strategy. A low target cell proportion typically implies a high concentration of background cells. Based on our findings (as shown in Figures 2, 4, 4f(i)), treating the background cells as the cells to be sorted results in a high recovery rate for these cells. This allows the majority of background cells to be removed in a single sorting step, significantly reducing their proportion in the mixture and naturally increasing the concentration ratio of target cells. Subsequently, a second round of sorting can be performed to sort the desired cells, resulting a higher recovery rate. In brief, for rare cell sorting, we employ a pre‐sorting step to remove background cells and increase the proportion of target cells, thereby improving the recovery rate.

To verify this strategy, we utilized a HeLa cells‐RBCs sample with a concentration ratio of ≈0.25 for HeLa cells, simulating a rare cell sorting scenario. Eight independent sorting each with a pre‐sorting step were performed. As shown in Table S9 and Figure S10 (Supporting Information), the number of background cells and the ratio of target cells were significantly altered after pre‐sorting. With a pre‐sorting cycle, we achieved recovery improvement of ≈30% (based on the simulation result in Figure 2d) for the “rare” HeLa cells.

**Figure 5:**
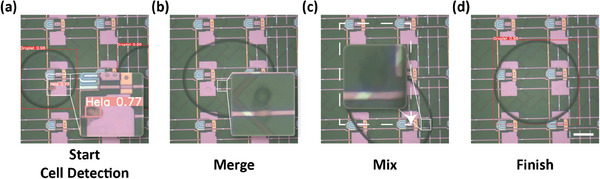
On‐chip cell lysis processing. (a) HeLa droplets were obtained by cell sorting and lysates were sampled. (b) Merge the two droplets. (c) The combined droplets are manipulated to circle and reciprocate so that their contents are well mixed and the lysis reaction is facilitated. (d) Lysis was completed and the object detection was performed, revealing no HeLa cells.

As AM‐DMF excels in droplet manipulation, we further developed a streamlined all‐in‐one cell sorting and manipulation strategy by performing on‐chip cell lysis. As shown in **Figure** **5**, after cell sorting, RIPA lysis buffer was introduced on‐chip and generated a single droplet, which was then merged with the HeLa cell droplet, mixed, and finally lysed the sorted cell. The entire process was automated, with the object detection on the droplets performed at the beginning and the lysis process performed at the end. HeLa cells were detected at the beginning and not at the end, confirming the lysis was effectively completed (as detailed in Movie S2, Supporting Information). This approach demonstrates great potential for downstream analysis of cells such as genomics and proteomics research in which cell lysis with minimum sample loss is the important first step and in great need.

**Figure 6:**
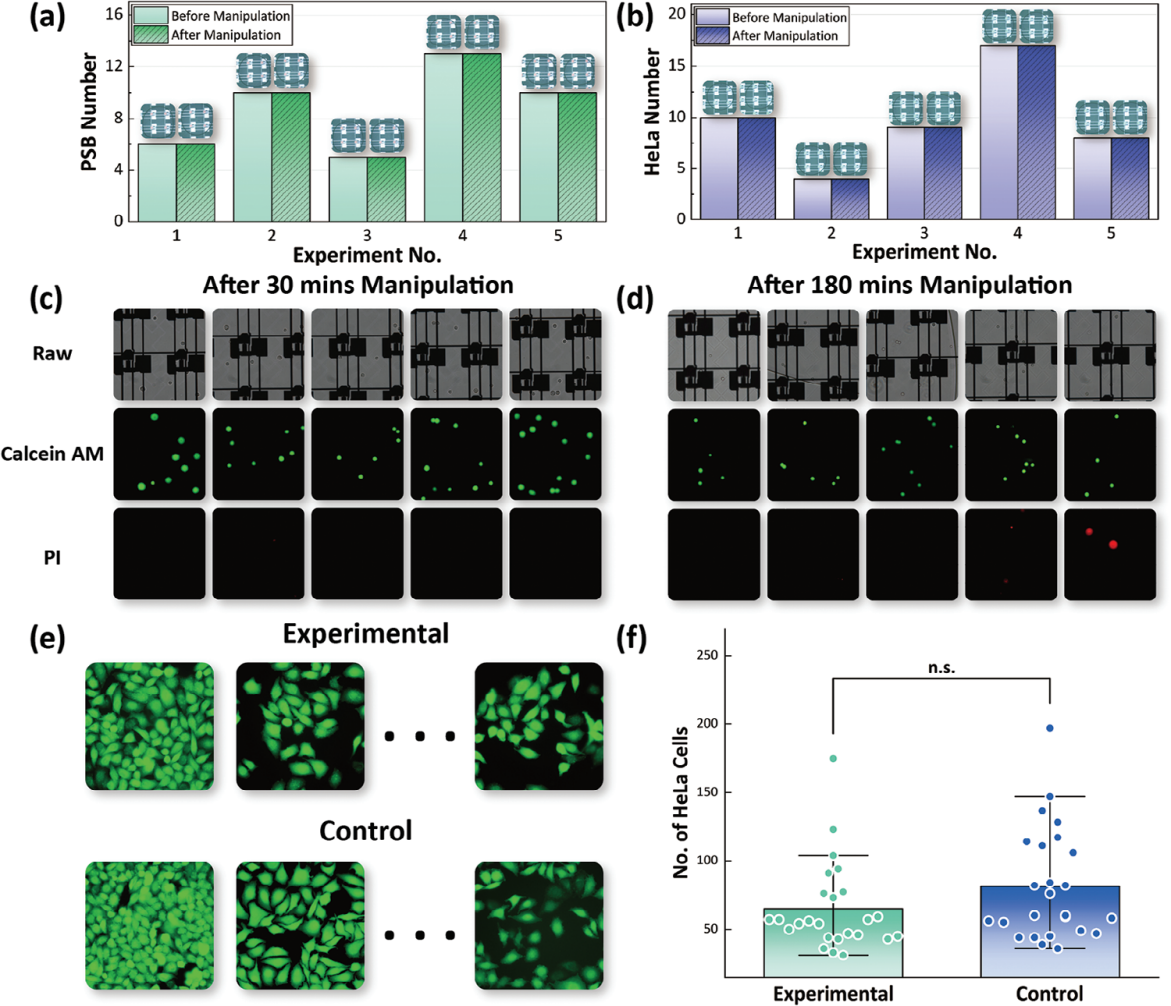
On‐chip and off‐chip assessment of the AM‐DMF manipulation on cell number loss and vitality. (a,b) On‐chip assessment of droplet manipulation's impact on cell loss for PSB and HeLa, respectively. (c,d) After 30 and 180 min of on‐chip manipulation, the HeLa cells were stained on‐chip and observed for vitality, respectively. (e) The experimental group was manipulated on‐chip for 30 min and then collected using capillary tubes, while the control group was collected with capillary tubes immediately after being injected on‐chip. Both groups were incubated in 48‐well plates for 24 h and subsequently stained. (f) The number of live cells in each group was counted in 48‐well plates, for which a paired *t*‐sample test was executed, and no significant differences were found.

Further, to verify that droplet manipulation on the AM‐DMF platform does not affect cell vitality and the number of carried cells, additional testing was conducted. We programmed an automated movement path and manipulated both PSBs and HeLa cells on the chip, allowing them to undergo 30 or 180 min of linear movement. The number and vitality of cells in each droplet before and after the movement were evaluated and plotted in **Figure** **6**. No changes were observed in the number and vitality of cells in the droplets before and after manipulation, confirming that no cell loss or damage occurred during droplet manipulation.

Finally, we investigated and compared a variety of microfluidic cell sorting methods,^[^ ^59^, ^60^, ^61^, ^62^, ^63^, ^64^, ^65^, ^66^, ^67^ ^]^ which are representative of different cell manipulation principles (e.g., surface acoustic wave, dielectrophoresis, optical tweezers, magnetic field, etc.) and different cell sorting devices, as shown in **Table** **1**. We also compared our method with several established machine learning‐assisted continuous flow cell sorting studies, as presented in Table S10 (Supporting Information). Compared to these alternatives, the cell sorting method proposed in this work uses a label‐free approach to achieve non‐contact manipulation of cells through the control of droplets, with each one function as a micro‐carrier for the cells. Although the throughput and sorting speed (8 groups of samples can be sorted simultaneously on one chip, with each group containing an average of 64 cells, within 2 min, resulting in a maximum sorting speed of 8 × 64/120 = 5 cells per s.) of our proposed method are somewhat limited by the hardware conditions (e.g., droplet moving speed, chip electrode size, etc.) of the AM‐DMF platform, the method offers several advantages over other techniques, such as high purity, cyclable sorting to enhance recovery, label‐free sorting, single‐cell analysis capability, and fully automatic sample preparation for integrated analysis. As a result, this approach is well‐suited for applications in single‐cell histology, single‐cell multi‐omics, and rare‐cell sorting.

**Table 1:** Comparison Between Different Cell Sorting Methods.

Method	Purity	Recovery	Label	Single‐cell analysis ability	Throughput	Sorting object	Refs.
This paper (3 cycles)	≈100%	≈80%	–	+	≈5 cell per s	HeLa Cells, PSBs, RBCs, Jurkat Cells, and HL‐60 cells	∖
Standing surface acoustic wave	92.3%	98.27%	+	–	1200 cells per s	HeLa cells	[59]
MEMS cavitation bubbles	95.8%	∖	+	–	23 000 cells per s	Euglena cell and gastric cancer cell	[60]
Traveling surface acoustic wave	>98%	98%	–	–	1000 cells per s	PSBs	[61]
Dielectrophoresis	>84%	67%–84%	–	–	90 cells per s	Human mesenchymal stem cells	[62]
Curved channel	98.4%	>90%	–	–	3 mL per min	CTCs and Blood Cells	[63]
Curved channel	91.4% WBCs 91.5% CTCs	89%–92.4%	–	–	2.5 mL per min	CTCs	[64]
Dean flow fractionation for size‐based separation	>90%	∖	–	–	0.13 mL per min	Neutrophils	[65]
Hydrodynamic filtration and magnetophoresis	90%	∖	+	–	100 cells per s	Human lymphocyte cells	[66]
Optical tweezers	89%	95%	+	–	1 cell per s	Yeast cells	[67]

## 3 Conclusion

In this study, we introduce a label‐free cell sorting technique that integrates deep learning image recognition with DMF manipulation to distinguish cells based on their morphological characteristics. By merging the automated parallel single‐cell operation of AM‐DMF with the rapid and precise object detection capabilities of the YOLOv8 algorithm, our method facilitates fully automated, high‐purity, and high‐recovery cell sorting with programmable nL droplets. The implementation of the safe‐interval path planning algorithm for droplet arrays ensures efficient spatial separation of droplets according to their contents, thereby enhancing the efficacy of cell sorting and the efficiency of the system. Extensive simulations and experiments were conducted to assess the influence of various parameters on on‐chip cell sorting. By establishing a mathematical model to approximate the Poisson distribution of droplet contents, we evaluated the impact of object detection model precision and PSB‐HeLa concentration ratios on sorting performance. Our results revealed a strong correlation between precision and purity, with the concentration ratio significantly affecting the recovery rate. Thus, our approach underscores the use of a highly accurate target recognition model, rendering it particularly suitable for sorting rare cells.

We further trained the YOLOv8 object detection model using images of droplets containing cells on the AM‐DMF chip, constructing a HeLa‐PSB dataset. To optimize model precision and achieve higher sorting recovery and purity, we compared three different versions of the YOLO model with varying parameter sizes, ultimately selecting YOLOv8s for its superior precision (98.5%) and short prediction time. Analysis of YOLOv8's training process, including the identification of suitable training epochs and validation using the confusion matrix, further confirmed its effectiveness. Additionally, we compared the diameter and circularity of HeLa cells and PSBs obtained through our approach with those from a cell counter. AM‐DMF's morphological analysis closely matched the actual distribution, highlighting the detection model's precision and accuracy. Differences in target morphology further underscored the model's high precision. We also examined the effect of the number of sorting cycles on purity and recovery, finding that 3–4 cycles were sufficient to achieve over 80% recovery and ≈100% purity at a 1:1 ratio. Similarly, we acquired images and constructed the HeLa‐RBC dataset using the same method and verified the object detection model's precision (97.52%) on it. Subsequently, we conducted several HeLa‐PSB, HeLa‐RBC, HeLa‐Jurkat, and HL‐60‐Jurkat sorting experiments on‐chip, achieving an average purity of 96.49%, 90.46%, 94.8%, and 95.2%, respectively, along with a three‐cycle average recovery of ≈80%. We then demonstrated the method's capability for all‐in‐one cell manipulation post‐sorting, exemplified by on‐chip single‐cell lysis. The experiences and system performance reported in this work are important to guide future users who would like to use such systems for cell sorting and related applications.

In conclusion, the deep learning‐assisted AM‐DMF method offers a robust label‐free cell sorting approach with high purity and high recovery rates. It shows potential in scenarios involving rare cells, such as CTC sorting. As a pioneering endeavor in DMF label‐free cell sorting, we anticipate further advancements and research efforts in this field, provide important life science tools for various biomedical and clinical applications, and continuously contributing to advancements in cell‐based diagnostics, drug development, and fundamental cell biology research.

## 4 Experimental Section

### AM‐DMF Platform for Cell Manipulation

The cell manipulation platform utilized in this study, known as DM sys, was engineered and assembled by ACX Instruments Ltd (Cambridge, U.K.) Engineered for fully automated and integrated single‐cell operations, DMF system can reach a droplet volume of 500 pL with an electrode dimension of 125 µm. Leveraging the “one‐to‐two” droplet splitting method introduced in prior research, DM sys can swiftly divide a droplet into 8 × 8 single‐cell droplet arrays within 10 s and execute massively parallel operations, laying the groundwork for subsequent cell sorting experiments.^[^ ^43^ ^]^ As depicted in Figure 1a (i), the system comprises three primary components: a motorized stage controlled by a triaxial motor, a camera equipped with high and low‐magnification lenses, and an AM‐DMF chip. Taking the AM16K chip as an example, it features 128 rows and 128 columns, with a side length of 4.5 cm, and was outfitted with injection holes on the upper glass substrate for the medium oil and samples, respectively. The AM‐DMF chip was composed of two glass substrates interposed with two hydrophobic layers, two insulation layers, and TFT pixel electrodes sandwiched in between.

### Object Detection Model for Cell Classification

The cutting‐edge YOLOv8 algorithm is employed as the object detection model in this research (https://github.com/ultralytics/ultralytics). YOLOv8, an abbreviation for “You Only Look Once version 8,” represents a significant advancement within the YOLO series of object detection models. Building upon the successes of its predecessors, YOLOv8 integrates advancements in deep learning methodologies to enhance real‐time object detection capabilities. Operationally, the YOLOv8 object detection model functions as follows: Initially, images of droplets containing cells on the AM‐DMF chip were acquired, pre‐processed, and subsequently fed into the YOLOv8 model for prediction. The model predicts Bboxes which were superimposed on the original images, yielding the desired output.

Regarding the training process, the initial step involved injecting a prepared suspension of HeLa cells mixed with PSB (for detailed preparation, refer to Section 2.4.) into the AM‐DMF chip. Subsequently, the “one‐to‐two” droplet splitting technique was employed to generate single droplet arrays. These droplet images were then captured using a 10× objective lens and uploaded to Roboflow (https://app.roboflow.com) for labeling, data pre‐processing (resized to 640 × 640 pixels in grayscale), and data augmentation (Table S4, Supporting Information). Given the uncertainty regarding the height of cells within the droplet, the model needed to possess the capability to accurately recognize cells at various heights. Therefore, for each droplet, three images were captured: one at the focal plane of the objective, another 10 µm above the focal plane, and the third 10 µm below the focal plane, as depicted in Figure 1a (iii). Following the initial screening process, a total of 163 images were curated to construct the dataset. Subsequent to data augmentation, the dataset expanded to 489 images, which were then partitioned into a training set (399 images, 82%), a validation set (60 images, 12%), and a test set (30 images, 6%) to form the HeLa‐PSB dataset. With image acquisition and data augmentation, the HeLa‐RBC dataset is established (609 images for training, 84 images for validation, and 56 images for testing), the HeLa‐Jurkat dataset (1350 images for training, 141 images for validation, and 95 images for testing), and the HL‐60 dataset (1185 images for training, 140 images for validation and 117 images for testing). All simulation experiments and model deployments were performed on a workstation equipped with an Intel Core i7‐12700H @3.70GHz × 20 CPUs, 32 GB RAM, and an NVIDIA RTX A2000 8GB Laptop GPU, utilizing Python 3.10.10. All Python scripts are available at https://github.com/Aneage/AM‐DMF‐Cell‐Sorting.

### Droplet Path Planning Algorithm

In this study, the SIPP^[^ ^68^ ^]^ is employed for the purpose of spatially segregating droplets according to their type to facilitate cell sorting. An issue encountered when applying SIPP to droplet path planning arises from its original design for unmanned vehicle path planning. In the context of unmanned vehicle path planning, two vehicles with a distance greater than 0 can be considered collision‐free. However, in droplet path planning, when two droplets were adjacent, surface tension causes them to merge, resulting in contamination and directly impacting the cell sorting outcome. To address this challenge, the safe distance between two droplets were set as 1 electrode and make corresponding modifications to the SIPP algorithm, and after planning the path for each droplet, a dynamic obstacle was set and get its safe interval according to the SIPP algorithm, thus realizing the collision‐free path planning for multiple droplets. Following the generation of the droplet array, droplets were identified using the object detection model, and their classifications were determined. The target electrodes for the respective droplets were then set, and the path‐planning algorithm was executed. (Algorithm 1, Supporting Information)

### Preparation of Samples

The HeLa cells were cultured under controlled conditions in a cell culture incubator set to 5% CO_2_, with saturated humidity, and maintained at 37 °C. The growth medium for the HeLa cells comprised MEM supplemented with 10% FBS, 100 µg mL^−1^ of penicillin, and 100 µg mL^−1^ of streptomycin (Eallbio, China). Prior to the commencement of experiments, the cells underwent dissociation and were resuspended in fresh complete medium. Both the cell count and cell viability were determined using an automated cell counter (Countess III, Thermo Fischer Scientific, USA). The cell lines were subcultured every 2–3 days at a density of 10^5^ cells mL^−1^ cm^−2^. Cell dissociation was facilitated by trypsin (Eallbio, China), followed by centrifugation at 800 rpm for 8 min. Subsequently, the supernatant was aspirated, and the cells were resuspended in a fresh complete medium.

The study, which involved clinical blood samples and data, was approved by the Ethics Committee of Peking Union Medical College Hospital (ethics number: I‐24PJ1716), and informed consent was obtained from all participants. Two milliliters of peripheral blood were collected in tubes, allowed to sit at room temperature for 1 h, and centrifuged at 100 g for 20 min. Aspirate the sample supernatant and resuspend the sample using PBS to obtain the red blood cell samples. Jurkat cells and HL‐60 cells were cultured under the same conditions as HeLa cells, using Jurkat complete medium (Eallbio, China) and HL‐60 complete medium (Eallbio, China), respectively. The cells were resuspended in PBS after centrifugation at 1050 rpm for 5 min.

The morphology of HeLa cells, PSBs, and RBCs differs greatly, allowing manual cell counting under a microscope. In contrast, HeLa, Jurkat, and HL‐60 cells have similar morphologies, with no visibly distinct differences. Therefore, the target cells were fluorescently labeled (live cells staining using Calcein‐AM, Phygene, China) prior to sorting, and were observed and counted under a fluorescence microscope after sorting. It was important to note that, although the target cells were stained, the fluorescent labeling information was used to differentiate between two types of cells to avoid manual counting errors due to their similarity in shape, not used to assist sorting, thereby maintaining the label‐free nature of the method.

The polystyrene beads (PSBs) utilized in the experiments possessed an average diameter of 10 µm, with a coefficient of variation of 10%, suspended in a 2.5% solids (w/v) aqueous solution, and a concentration of 4.55 × 10^7^ particles per mL (Polybead® Microspheres 10.00 µm, Polysciences, U.S.A.). Prior to experimentation, the PSB was diluted 100‐fold using PBS buffer (Eallbio, China) and mixed with HeLa cells in an equal volume ratio. To ensure stable on‐chip manipulation upon injection onto the AM‐DMF chip, a 0.05% (v/v) P‐F68 surfactant (Poloxamer 188, Sigma‐Aldrich, Germany) was added to the HeLa/PSB mixed suspension. The concentration of all the samples in the sorting experiments were obtained from the cell counter, while the number of particles used to calculate the recovery and purity were manually counted to ensure accuracy.

### Evaluation of Droplet Manipulation on Cells

To assess potential cell loss or damage during droplet manipulation on the AM‐DMF chip, the following experiments were performed: HeLa cells and PSB samples were injected into the chip, automated movement paths were programmed for the droplets, and the droplets were manipulated for 30 min. The number of cells contained in each droplet was counted both before and after the manipulation and was plotted in Figure 6a,b. To assess cellular vitality and functionality after sorting, two tests were conducted. First, the cells are automatically manipulated on‐chip for 30 min and 180 min, respectively, and then loaded fluorescent dye solution on‐chip for staining to differentiate between live and dead cells under a fluorescence microscope. The results in Figure 6c,d show that no changes in cell vitality were observed after the 30‐min manipulation, while cell vitality slightly decreased after the 180‐min manipulation, indicated by a weaker fluorescent signal.

Furthermore, the cell samples were divided into experimental and control groups. The experimental group was manipulated on‐chip for 30 min and then collected using capillary tubes, while the control group was collected with capillary tubes immediately after being injected on‐chip. Both groups were incubated in 48‐well plates for 24 h and subsequently stained. The number of live cells in each group was counted and shown in Figure 6e,f. A paired samples *t*‐test revealed no significant difference in the number of live cells between the two groups, indicating that cell viability and functionality were not affected by the AM‐DMF operation. This demonstrates that the proposed method maintains cellular vitality well during sorting.

Integrating the methodologies mentioned above and procedures yields the comprehensive process of cell sorting on the AM‐DMF platform, as illustrated in Figure 1. Initially, the sample was injected into the AM‐DMF chip, where the droplet array was generated via the execution of the “one‐to‐two” droplet splitting method. Subsequently, the droplet array was screened under the field of view of a 10× objective lens to get droplet classification data using the YOLOv8 object detection model. Following this, the droplet path planning algorithm was employed to segregate and rearrange the droplets according to their classification. Finally, the sorting outcomes were analyzed to obtain information such as purity and recovery rate. Notably, the whole process can be iterated to enhance the recovery rate. After segregating the HeLa droplets, the necessary droplets required to reconstitute an 8 × 8 array were calculated, and samples (fresh droplets) were added. The remaining droplets were then combined with the fresh droplets, and the process of splitting and sorting was repeated. This iterative procedure facilitates the separation of the two target types within the mixed droplets, thereby improving the recovery rate (Movie S1, **Algorithm 2**, Supporting Information).

## Conflict of Interest

The authors declare no conflict of interest.

## Supporting information



Supporting Information

Supplemental Movie 1

Supplemental Movie 2

## Data Availability

The data that support the findings of this study are available from the corresponding author upon reasonable request.
