# Supplementary material for: Deep Learning‐Assisted Label‐Free Parallel Cell Sorting with Digital Microfluidics
Source: Adv Sci (Weinh). 2024 Nov 5;12(1):2408353. doi: 10.1002/advs.202408353 (PMC11906218; doi:10.1002/advs.202408353)
Supplement: Supplementary file 1 — Supporting Information [file ADVS-12-2408353-s002.docx]

Supporting Information

**Deep Learning-assisted Label-free Parallel Cell Sorting with Digital Microfluidics**

*Zongliang Guo, Fenggang Li, Hang Li*, Menglei Zhao, Haobing Liu, Haopu Wang, Hanqi Hu, Rongxin Fu, Yao Lu, Siyi Hu, Huikai Xie, Hanbin Ma*, and Shuailong Zhang**

Z. Guo, F. Li, M. Zhao, H. Liu, S. Zhang

Beijing Advanced Innovation Center for Intelligent Robots and Systems,

Beijing Institute of Technology, Beijing 100081, China

*E-mail: shuailong.zhang@bit.edu.cn

H. Hu, R. Fu, H. Li

School of Medical Technology,

Beijing Institute of Technology, Beijing 100081, China
*E-mail: hang.li@bit.edu.cn

H. Wang, Y. Lu, H. Xie, S. Zhang

School of Integrated Circuits and Electronics, Engineering Research Center of Integrated Acousto-opto-electronic Microsystems (Ministry of Education of China), Beijing Institute of Technology, Beijing 100081, China

S. Hu, H. Ma

CAS Key Laboratory of Bio-Medical Diagnostics, Suzhou Institute of Biomedical Engineering and Technology, Chinese Academy of Sciences, Suzhou 215163, China

ACX Instruments Ltd, St John’s Innovation Centre, Cambridge CB40WS, U.K.

*E-mail: mahb@sibet.ac.cn

**Mathematical derivation of the simulation model for cell sorting**

To simply the analysis, precision is defined as the average of the model’s prediction accuracies for HeLa cells and for PSBs.

$$\begin{aligned} Recovery=\frac{n_{h}}{N_{h}}\#\left( 1 \right) \end{aligned}$$

$$\begin{aligned} Purity=\frac{n_{h}}{n_{h}+n_{p}}\#\left( 2 \right) \end{aligned}$$

$$\begin{aligned} Precision=\frac{TP}{TP+FP}\#\left( 3 \right) \end{aligned}$$

$$\begin{aligned} Ratio=\frac{c_{h}}{c_{p}}\#\left( 4 \right) \end{aligned}$$

In which, $n_{h}$ is the number of sorted HeLa cells in the sorting result, while $N_{h}$ indicates the number of HeLa cells prior to sorting. Similarly, $n_{p}$ represents the number of sorted PSBs. The terms $c_{p}$ and $c_{p}$ refer to the concentrations of PSBs and HeLa cells, separately. Additionally, $TP$ and $FP$ are abbreviations for true positive and false positive respectively.

Assuming that the cells in the sample are spatially uniformly distributed and that the "one-to-two" droplet segmentation method is symmetric for each segmentation of the droplet. When the concentration of the sample is $3.2\times{10}^{5}/mL$, the number of HeLa cells ($x_{hi}$) and PSBs ($x_{pi}$) in each droplet obeys the following distribution:

$$x_{hi}\sim P\left( \lambda=1 \right)$$

$$x_{pi}\sim P(\lambda=r)$$

where $r$ is the concentration ratio of PSBs to HeLa cells, $i=1,2,...,n$, $n$ is the number of droplets obtained in a single load of sample.

Here, we use $d_{i}=\left( x_{hi},x_{pi} \right)$ to represent the actual number of HeLa cells and PSBs contained in the $i$th droplet, and $\tilde{d}_{i}=\left( y_{hi},x_{pi} \right)$ (since the total number of objects in the droplet remains unchanged during the cell sorting) to represent the recognition result of the object detection model for the $i$th droplet, $y_{hi}=x_{hi}^{T}+x_{pi}^{F}$, i.e., the predicted number of HeLa cells in the $i$th droplet is the sum of the number of correctly classified HeLa cells and the number of misclassified PSBs and similarly $y_{pi}$. If the model precision is $p$, i.e., for each object, there is a probability of $p$ that it will be classified correctly, then the probability distribution of $x_{hi}^{T}$ is:

$$\begin{matrix} & P\left( x_{hi}^{T}=k \right)=C_{x_{hi}}^{k}p^{k}\left( 1-p \right)^{x_{hi}-k} \end{matrix}$$

The same method can be used to derive the probability distribution of $x_{pi}^{F}$. Then the probability distribution of $y_{hi}$ is the joint distribution of $x_{hi}^{T}$ and $x_{pi}^{F}$, i.e., the probability mass function of $y_{hi}$ is the convolution of the $x_{hi}^{T}$ and $x_{pi}^{F}$ probability mass functions:

$$\begin{matrix} & P\left( y_{hi}=y \right)=\sum_{k=0}^{y} P\left( x_{hi}^{T}=k \right)P\left( x_{pi}^{F}=y-k \right) \end{matrix}$$

Then, the probability distribution of the classification result $c_{i}$ for the $i$th droplet is:

$$P\left( c_{i}=HeLa \right)=P\left( y_{hi}=x_{hi}+x_{pi} \right)$$

$$=P\left( x_{hi}^{T}= x_{hi}, x_{pi}^{F}= x_{pi} \right)$$

$$P\left( c_{i}=PSB \right)=P\left( y_{hi}=0 \right)$$

$$=P\left( x_{hi}^{T}= 0, x_{pi}^{F}= 0 \right)$$

$$P\left( c_{i}=Mixed \right)=P\left( 0<y_{hi}<x_{hi}+x_{pi} \right)$$

$$=P\left( 0<x_{hi}^{T}+x_{pi}^{F}<x_{hi}+x_{pi} \right)$$

$$P\left( c_{i}=Empty \right)=P\left( y_{hi}=0,x_{hi}+x_{pi}-y_{hi}=0 \right)$$

$$=P\left( x_{hi}=0,x_{pi}=0 \right)$$

When the target cells are HeLa cells, the droplets with the classification of $c_{i}=HeLa$ will be sorted out, and the remaining droplets will be left in situ waiting for the next cycle, so $n_{h}$, $n_{p}$, and $N_{h}$ are calculated as follows:

$$n_{h}= \sum_{c_{i}=HeLa} x_{hi}$$

$$n_{p}= \sum_{c_{i}=HeLa} x_{pi}$$

$$N_{h}= \sum_{i} x_{hi}$$

and from which recoveries and purities can be calculated.

**Supplementary Table**

**Table S1.** Comparison Between Different Cell Sorting Methods.

| **Method** | **Throughput** | **Repeated Imaging** | **Spatial Resolution** | **Label** | **Refs** |
| --- | --- | --- | --- | --- | --- |
| FACS | ~4000 (cells/s) | - | \ | + | [12-15] |
| MACS | ~2.38 ($\mu$L/s) | - | \ | + | [17-18] |
| Microfluidic IBCS | 100~3000 (cells/s) | + | Low | - | [20-23, 25] |
| Microarray  IBCS | 1~10 (cells/s) | + | High | - | [24, 26] |

**Table S2.** Details for Different Models.

| **Model** | | **Year** | **Parameter Size (M)** |
| --- | --- | --- | --- |
| YOLOv5 | Nano | 2020 | 1.9 |
|  | Small |  | 7.2 |
|  | Middle |  | 21.2 |
|  | Large |  | 46.5 |
| YOLOv7 | Tiny | 2022 | 6.2 |
|  | Middle |  | 36.9 |
|  | Large |  | 71.3 |
| YOLOv8 | Nano | 2023 | 3.2 |
|  | Small |  | 11.2 |
|  | Middle |  | 25.9 |
|  | Large |  | 43.7 |

**Table S3.** Distribution of No. of PSBs in droplets at different $\lambda$.

| **No. of PSBs** | **1.021875** | **2.0625** | **2.453125** |
| --- | --- | --- | --- |
| 0 | 18 | 11 | 13 |
| 1 | 20 | 8 | 10 |
| 2 | 11 | 22 | 18 |
| 3 | 6 | 10 | 12 |
| 4 | 9 | 4 | 5 |
| 5 | 0 | 2 | 1 |
| 6 | 0 | 1 | 1 |
| 7 | 0 | 6 | 4 |

**Table S4.** Parameter settings for data augmentation

| **Parameter** | **Setting** |
| --- | --- |
| Brightness | Between -25% and +25% |
| Exposure | Between -10% and +10% |
| Blur | Up to 2px |
| Noise | Up to 2% of pixels |
| Bounding Box Brightness | Between -10% and +10% |
| Bounding Box Exposure | Between -10% and +10% |
| Bounding Box Blur | Up to 2px |
| Bounding Box Noise | Up to 5% of pixels |

**Table S5.** Experiment Results on Hela-PSB Mixture

| **Concentration HeLa Cell (×10^5/mL)** | **Concentration PSB (×10^5/mL)** | **Ratio** | **Purity** | **Recovery** | **Cycle** |
| --- | --- | --- | --- | --- | --- |
| 1.34 | 3.88 | 0.35 | 100.00% | 24% | 1 |
|  |  |  | 100.00% | 28% | 2 |
|  |  |  | 88.89% | 36% | 3 |
| 1.01 | 2.80 | 0.36 | 100.00% | 11.11% | 1 |
|  |  |  | 100.00% | 33.33% | 2 |
|  |  |  | 100.00% | 44.44% | 3 |
| 1.51 | 3.13 | 0.48 | 100.00% | 22.73% | 1 |
|  |  |  | 100.00% | 31.82% | 2 |
|  |  |  | 90.00% | 45.45% | 3 |
| 1.39 | 2.15 | 0.65 | 100.00% | 29.03% | 1 |
|  |  |  | 92.31% | 41.94% | 2 |
|  |  |  | 95.00% | 64.51% | 3 |
| 1.07 | 1.61 | 0.67 | 100.00% | 26.67% | 1 |
|  |  |  | 85.71% | 46.67% | 2 |
|  |  |  | 90.91% | 73.33% | 3 |
| 1.82 | 2.55 | 0.71 | 100.00% | 23.08% | 1 |
|  |  |  | 100.00% | 53.85% | 2 |
|  |  |  | 94.74% | 73.08% | 3 |
| 1.49 | 1.99 | 0.75 | 100.00% | 30.77% | 1 |
|  |  |  | 100.00% | 42.31% | 2 |
|  |  |  | 94.12% | 65.38% | 3 |
| 1.65 | 2.15 | 0.77 | 100.00% | 31.58% | 1 |
|  |  |  | 100.00% | 47.37% | 2 |
|  |  |  | 92.86% | 73.68% | 3 |
| 1.17 | 1.32 | 0.88 | 100.00% | 29.41% | 1 |
|  |  |  | 100.00% | 41.18% | 2 |
|  |  |  | 90.91% | 64.71% | 3 |
| 1.20 | 1.26 | 0.96 | 100.00% | 22.22% | 1 |
|  |  |  | 100.00% | 44.44% | 2 |
|  |  |  | 92.86% | 77.78% | 3 |
| 4.15 | 4.00 | 1.04 | 95.83% | 32.88% | 1 |
|  |  |  | 97.14% | 47.95% | 2 |
|  |  |  | 98.25% | 78.08% | 3 |
| 3.93 | 3.50 | 1.12 | 100.00% | 34.78% | 1 |
|  |  |  | 96.97% | 47.83% | 2 |
|  |  |  | 98.15% | 78.26% | 3 |
| 1.49 | 1.24 | 1.20 | 100.00% | 38.89% | 1 |
|  |  |  | 90.91% | 61.11% | 2 |
|  |  |  | 93.75% | 88.89% | 3 |
| 3.03 | 2.32 | 1.31 | 86.67% | 36.59% | 1 |
|  |  |  | 92.59% | 65.85% | 2 |
|  |  |  | 93.75% | 78.05% | 3 |
| 4.88 | 3.73 | 1.31 | 100.00% | 33.77% | 1 |
|  |  |  | 97.44% | 50.65% | 2 |
|  |  |  | 98.33% | 77.92% | 3 |
| 1.52 | 1.11 | 1.36 | 87.50% | 38.10% | 1 |
|  |  |  | 90.91% | 52.38% | 2 |
|  |  |  | 80.00% | 71.43% | 3 |
| 1.41 | 0.98 | 1.44 | 100.00% | 42.11% | 1 |
|  |  |  | 100.00% | 57.89% | 2 |
|  |  |  | 100.00% | 84.21% | 3 |
| 1.54 | 0.85 | 1.81 | 100.00% | 44.44% | 1 |
|  |  |  | 100.00% | 66.67% | 2 |
|  |  |  | 100.00% | 92.59% | 3 |
| 4.43 | 2.34 | 1.89 | 97.14% | 49.30% | 1 |
|  |  |  | 98.04% | 71.83% | 2 |
|  |  |  | 98.41% | 88.73% | 3 |

**Table S6.** Experiment Results on Hela-RBC Mixture

| **Concentration HeLa Cell (×10^5/mL)** | **Concentration RBC (×10^5/mL)** | **Ratio** | **Purity** | **Recovery** | **Cycle** |
| --- | --- | --- | --- | --- | --- |
| 2.93 | 4.98 | 0.59 | 76.92% | 23.64% | 1 |
|  |  |  | 82.35% | 30.91% | 2 |
|  |  |  | 80.00% | 45.45% | 3 |
| 1.66 | 2.78 | 0.60 | 80.00% | 21.74% | 1 |
|  |  |  | 85.71% | 30.43% | 2 |
|  |  |  | 80.00% | 43.48% | 3 |
| 1.56 | 2.12 | 0.74 | 100.00% | 30.77% | 1 |
|  |  |  | 100.00% | 30.77% | 2 |
|  |  |  | 100.00% | 53.85% | 3 |
| 4.73 | 5.18 | 0.91 | 82.35% | 25.76% | 1 |
|  |  |  | 87.10% | 46.97% | 2 |
|  |  |  | 91.67% | 72.73% | 3 |
| 5.08 | 5.10 | 1.00 | 92.31% | 32.91% | 1 |
|  |  |  | 93.18% | 51.90% | 2 |
|  |  |  | 94.12% | 78.48% | 3 |
| 3.25 | 3.20 | 1.02 | 95.45% | 37.29% | 1 |
|  |  |  | 96.77% | 52.54% | 2 |
|  |  |  | 92.00% | 84.75% | 3 |
| 2.75 | 2.63 | 1.05 | 94.44% | 31.58% | 1 |
|  |  |  | 93.94% | 57.89% | 2 |
|  |  |  | 93.33% | 78.95% | 3 |
| 2.58 | 2.25 | 1.14 | 88.89% | 32.73% | 1 |
|  |  |  | 89.66% | 52.73% | 2 |
|  |  |  | 87.18% | 70.91% | 3 |
| 5.78 | 4.95 | 1.17 | 82.35% | 40.48% | 1 |
|  |  |  | 86.96% | 54.76% | 2 |
|  |  |  | 87.30% | 75.00% | 3 |
| 1.89 | 1.62 | 1.17 | 87.50% | 35.71% | 1 |
|  |  |  | 90.00% | 50.00% | 2 |
|  |  |  | 90.91% | 78.57% | 3 |
| 3.15 | 2.60 | 1.21 | 89.47% | 30.16% | 1 |
|  |  |  | 94.29% | 55.56% | 2 |
|  |  |  | 91.67% | 76.19% | 3 |
| 2.19 | 1.53 | 1.43 | 88.24% | 34.69% | 1 |
|  |  |  | 88.00% | 51.02% | 2 |
|  |  |  | 90.00% | 81.63% | 3 |
| 3.35 | 2.34 | 1.43 | 100.00% | 37.04% | 1 |
|  |  |  | 96.55% | 53.70% | 2 |
|  |  |  | 97.56% | 75.93% | 3 |
| 3.43 | 2.21 | 1.55 | 82.76% | 42.65% | 1 |
|  |  |  | 88.10% | 61.76% | 2 |
|  |  |  | 90.00% | 88.24% | 3 |
| 2.60 | 1.52 | 1.71 | 96.43% | 46.67% | 1 |
|  |  |  | 91.89% | 61.67% | 2 |
|  |  |  | 94.12% | 85.00% | 3 |
| 3.08 | 1.40 | 2.20 | 92.59% | 45.76% | 1 |
|  |  |  | 95.12% | 69.49% | 2 |
|  |  |  | 92.86% | 94.92% | 3 |

**Table S7.** Label-free HeLa-Jurkat cell sorting using the deep learning-assisted DMF system.

| **Concentration HL-60 (×10^5^/mL)** | **Concentration Jurkat (×10^5^/mL)** | **Ratio** | **Purity** | **Recovery** | **Cycle** |
| --- | --- | --- | --- | --- | --- |
| 2.67 | 0.94 | 2.84 | 100.00% | 68.42% | 1 |
|  |  |  | 97.78% | 78.95% | 2 |
|  |  |  | 97.92% | 84.21% | 3 |
| 2.27 | 0.99 | 2.29 | 100.00% | 71.43% | 1 |
|  |  |  | 97.73% | 78.57% | 2 |
|  |  |  | 98.08% | 92.86% | 3 |
| 2.04 | 1.05 | 1.94 | 100.00% | 61.22% | 1 |
|  |  |  | 97.06% | 69.39% | 2 |
|  |  |  | 97.50% | 93.88% | 3 |
| 1.99 | 1.26 | 1.57 | 95.45% | 45.83% | 1 |
|  |  |  | 96.77% | 64.58% | 2 |
|  |  |  | 94.87% | 87.50% | 3 |
| 1.36 | 1.28 | 1.06 | 100.00% | 35.14% | 1 |
|  |  |  | 94.74% | 51.35% | 2 |
|  |  |  | 93.10% | 78.38% | 3 |
| 1.38 | 1.39 | 0.99 | 90.91% | 28.21% | 1 |
|  |  |  | 94.44% | 46.15% | 2 |
|  |  |  | 96.97% | 84.62% | 3 |
| 1.34 | 1.42 | 0.94 | 100.00% | 31.71% | 1 |
|  |  |  | 94.74% | 46.34% | 2 |
|  |  |  | 96.77% | 75.61% | 3 |
| 1.31 | 1.51 | 0.86 | 100.00% | 32.35% | 1 |
|  |  |  | 100.00% | 55.88% | 2 |
|  |  |  | 92.00% | 73.53% | 3 |
| 1.26 | 1.59 | 0.79 | 100.00% | 31.43% | 1 |
|  |  |  | 100.00% | 51.43% | 2 |
|  |  |  | 95.65% | 65.71% | 3 |
| 1.23 | 1.65 | 0.74 | 100.00% | 41.94% | 1 |
|  |  |  | 100.00% | 51.61% | 2 |
|  |  |  | 94.74% | 61.29% | 3 |
| 1.17 | 1.73 | 0.67 | 100.00% | 28.13% | 1 |
|  |  |  | 93.33% | 46.88% | 2 |
|  |  |  | 94.74% | 59.38% | 3 |
| 1.15 | 1.76 | 0.65 | 100.00% | 31.03% | 1 |
|  |  |  | 100.00% | 48.28% | 2 |
|  |  |  | 93.75% | 55.17% | 3 |
| 1.15 | 1.78 | 0.64 | 100.00% | 23.53% | 1 |
|  |  |  | 100.00% | 44.12% | 2 |
|  |  |  | 94.44% | 52.94% | 3 |
| 1.1 | 1.81 | 0.60 | 87.50% | 28.57% | 1 |
|  |  |  | 83.33% | 42.86% | 2 |
|  |  |  | 80.00% | 53.57% | 3 |
| 1.08 | 1.85 | 0.58 | 85.71% | 25.93% | 1 |
|  |  |  | 90.00% | 37.04% | 2 |
|  |  |  | 92.86% | 51.85% | 3 |
| 1.02 | 1.94 | 0.52 | 100.00% | 28.00% | 1 |
|  |  |  | 100.00% | 40.00% | 2 |
|  |  |  | 91.67% | 48.00% | 3 |
| 0.97 | 2.01 | 0.48 | 80.00% | 27.78% | 1 |
|  |  |  | 83.33% | 33.33% | 2 |
|  |  |  | 87.50% | 44.44% | 3 |
| 0.95 | 2.04 | 0.46 | 100.00% | 33.33% | 1 |
|  |  |  | 88.89% | 42.86% | 2 |
|  |  |  | 90.91% | 52.38% | 3 |
| 0.92 | 2.07 | 0.44 | 100.00% | 9.09% | 1 |
|  |  |  | 100.00% | 22.73% | 2 |
|  |  |  | 87.50% | 36.36% | 3 |
| 1.01 | 2.15 | 0.46 | 100.00% | 14.29% | 1 |
|  |  |  | 100.00% | 23.81% | 2 |
|  |  |  | 88.89% | 42.86% | 3 |

**Table S8.** Label-free HL-60-Jurkat cell sorting using the deep learning-assisted DMF system.

| **Concentration HL-60 (×10^5^/mL)** | **Concentration Jurkat (×10^5^/mL)** | **Ratio** | **Purity** | **Recovery** | **Cycle** |
| --- | --- | --- | --- | --- | --- |
| 2.25 | 3.31 | 0.68 | 100.00% | 23.91% | 1 |
|  |  |  | 100.00% | 56.52% | 2 |
|  |  |  | 96.88% | 69.57% | 3 |
| 7.90 | 1.85 | 4.27 | 96.91% | 68.31% | 1 |
|  |  |  | 97.39% | 80.99% | 2 |
|  |  |  | 96.18% | 92.25% | 3 |
| 7.55 | 1.94 | 3.89 | 96.88% | 60.38% | 1 |
|  |  |  | 94.40% | 78.62% | 2 |
|  |  |  | 93.62% | 88.68% | 3 |
| 2.96 | 2.51 | 1.18 | 100.00% | 29.09% | 1 |
|  |  |  | 96.55% | 43.64% | 2 |
|  |  |  | 95.00% | 52.73% | 3 |
| 9.00 | 1.81 | 4.97 | 91.67% | 78.50% | 1 |
|  |  |  | 90.77% | 91.12% | 2 |
|  |  |  | 91.22% | 95.79% | 3 |
| 6.05 | 1.51 | 4.01 | 91.25% | 66.12% | 1 |
|  |  |  | 90.82% | 80.99% | 2 |
|  |  |  | 90.91% | 90.91% | 3 |
| 5.68 | 1.59 | 3.57 | 98.44% | 65.98% | 1 |
|  |  |  | 93.42% | 78.35% | 2 |
|  |  |  | 93.83% | 83.51% | 3 |
| 4.68 | 1.76 | 2.66 | 98.31% | 67.82% | 1 |
|  |  |  | 98.48% | 75.86% | 2 |
|  |  |  | 93.33% | 86.21% | 3 |
| 4.72 | 1.73 | 2.73 | 93.33% | 73.17% | 1 |
|  |  |  | 93.94% | 80.49% | 2 |
|  |  |  | 94.37% | 86.59% | 3 |
| 5.20 | 1.65 | 3.15 | 97.14% | 67.31% | 1 |
|  |  |  | 92.11% | 73.08% | 2 |
|  |  |  | 90.24% | 78.85% | 3 |
| 4.50 | 2.01 | 2.24 | 97.96% | 53.85% | 1 |
|  |  |  | 87.72% | 62.64% | 2 |
|  |  |  | 85.94% | 70.33% | 3 |
| 3.61 | 2.04 | 1.77 | 100.00% | 46.58% | 1 |
|  |  |  | 90.24% | 56.16% | 2 |
|  |  |  | 91.67% | 82.19% | 3 |
| 3.48 | 2.07 | 1.68 | 100.00% | 46.97% | 1 |
|  |  |  | 97.73% | 66.67% | 2 |
|  |  |  | 90.38% | 78.79% | 3 |
| 3.45 | 2.15 | 1.60 | 100.00% | 57.63% | 1 |
|  |  |  | 95.00% | 67.80% | 2 |
|  |  |  | 93.48% | 77.97% | 3 |
| 3.06 | 2.17 | 1.41 | 96.55% | 48.33% | 1 |
|  |  |  | 97.06% | 56.67% | 2 |
|  |  |  | 90.91% | 73.33% | 3 |
| 7.05 | 1.39 | 5.07 | 93.75% | 76.71% | 1 |
|  |  |  | 93.33% | 92.47% | 2 |
|  |  |  | 89.44% | 97.26% | 3 |
| 2.67 | 2.59 | 1.03 | 92.86% | 24.14% | 1 |
|  |  |  | 95.00% | 34.48% | 2 |
|  |  |  | 90.91% | 56.90% | 3 |
| 2.59 | 3.03 | 0.85 | 100.00% | 31.15% | 1 |
|  |  |  | 96.00% | 40.98% | 2 |
|  |  |  | 96.97% | 54.10% | 3 |
| 2.46 | 3.16 | 0.78 | 100.00% | 23.08% | 1 |
|  |  |  | 94.74% | 36.54% | 2 |
|  |  |  | 95.65% | 44.23% | 3 |
| 2.44 | 3.32 | 0.73 | 100.00% | 34.55% | 1 |
|  |  |  | 97.06% | 61.82% | 2 |
|  |  |  | 97.56% | 74.55% | 3 |
| 6.95 | 1.42 | 4.89 | 93.44% | 79.74% | 1 |
|  |  |  | 92.54% | 87.58% | 2 |
|  |  |  | 91.03% | 94.77% | 3 |

**Table S9.** Experiment Results on Pre-sorting of Hela-RBC

| **Experiment No.** | | **No. of HeLa** | **No. of RBC** | **Ratio (HeLa/RBC)** | **RBC Sorted** |
| --- | --- | --- | --- | --- | --- |
| 1 | Pre Sorting | 13 | 68 | 0.19 | 57 |
|  | Sorting |  | 11 | 1.18 |  |
| 2 | Pre Sorting | 19 | 56 | 0.34 | 38 |
|  | Sorting |  | 18 | 1.06 |  |
| 3 | Pre Sorting | 11 | 70 | 0.16 | 62 |
|  | Sorting |  | 8 | 1.38 |  |
| 4 | Pre Sorting | 18 | 77 | 0.23 | 51 |
|  | Sorting |  | 10 | 1.80 |  |
| 5 | Pre Sorting | 24 | 62 | 0.39 | 43 |
|  | Sorting |  | 19 | 1.26 |  |
| 6 | Pre Sorting | 16 | 82 | 0.20 | 71 |
|  | Sorting |  | 11 | 1.45 |  |
| 7 | Pre Sorting | 15 | 65 | 0.23 | 53 |
|  | Sorting |  | 12 | 1.25 |  |
| 8 | Pre Sorting | 21 | 86 | 0.24 | 72 |
|  | Sorting |  | 14 | 1.50 |  |

**Table S10.** Comparison with other machine learning-assisted continuous flow cell sorting works.

| **Ref** | **Recovery** | **Purity** | **Single Cell Sorting and Manipulation** | **Main Method** |
| --- | --- | --- | --- | --- |
| This Work | ~80% | ~100% | + | YOLOv8  AM-DMF |
| [20] | 72.7% | 99.1% | - | Deep Convolutional Neural Network  Microfluidic |
| [21] | \ | 95.1% | - | Ghost Imaging  Supporting Vector Machine |
| [25] | 55.5%-75.9% | 94.2%-95.1% | - | TensorRT Framework  Microfluidic |
| [27] | \ | 89.7% | - | Surface Acoustic Waves  Deep Neural Network |

| **Algorithm 1:** Safe Interval Path Planning Algorithm For Multiple Droplets |
| --- |
| % Initialize the map of multiple droplets path planning. %  map=initialize(agents=droplets,  starts=droplets,  goals=greedy_assign_goals(droplets),  dynamic_obstacles = [])  for droplet in droplets:  path = sipp_planner(map, droplet)  paths.update(path) |
| % Avoid collisions by treating each droplet with a planned path as a dynamic obstacle.  map.dynamic_obstacles.update(droplet) |
| return paths  sipp_planner(map, droplet):  % Initialize, heuristic() is the heuristic cost function, and cost = 1 as droplets move in 4 directions.%  open = [], g = 0, f(starts) = heuristic(map.starts), cost = 1  open.append(f(starts))  while (goal not reached):  s = open.pop()  successors = get_successors(s)  for successor in successors:  if g(successor) > g(s) + cost:  g(successor) = g(s) + cost  successor.parent_state = s  if successor = goal: goal reached.  successor.f = successor.g + heuristic(successor.position)  open.append(successor) |

| **Algorithm 2:** Cell Sorting Algorithm |
| --- |
| % Stage 1: Capture and classify droplet images at high magnification. %  model = load_model(“yolov8_cell_sorting.pt”)  droplets = []  for i in range (64):  % Automatically move the triaxial motor to the position of the ith droplet and take picture.%  img = capture_img(i)  classes = model.predict(img).boxes.cls  if ‘HeLa’ in classes and ‘PSB’ in classes:  droplets.append(‘Mixed’)  else if ‘HeLa’ in classes:  droplets.append(‘HeLa’)  else if ‘PSB’ in classes:  droplets.append(‘PSB’)  else:  droplets.append(‘Empty’) |
| % Stage 2: Path planning according to the classification results. % |
| paths = SIPP(droplets)  execute(paths) |


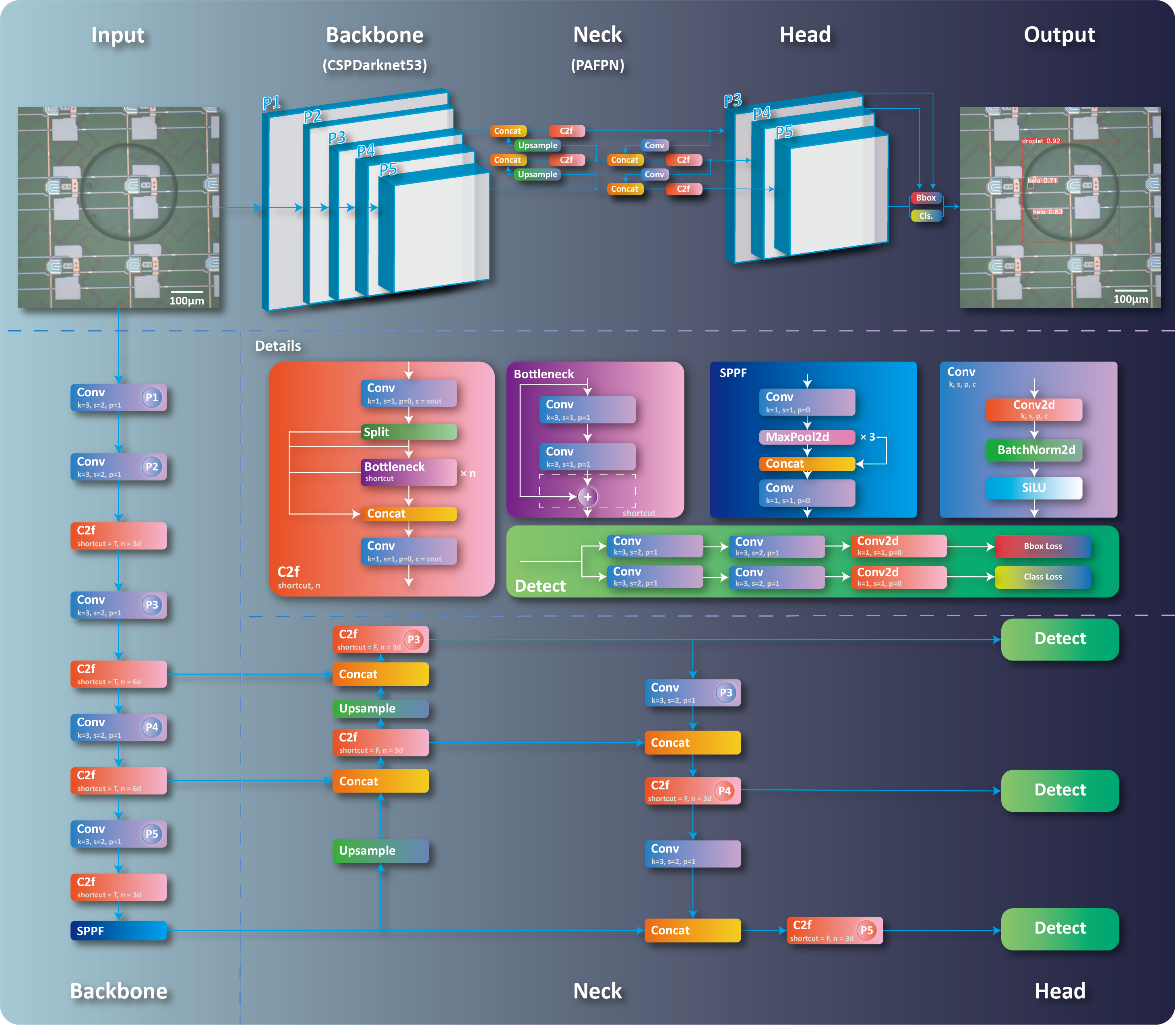


**Figure S1.** The Structure and Prediction Workflow of YOLOv8. YOLOv8 network structure is mainly divided into three parts: Backbone, Neck, and Head. The preprocessed droplet image is predicted by the network to get the classification information and Bboxes and plotted on the original image.


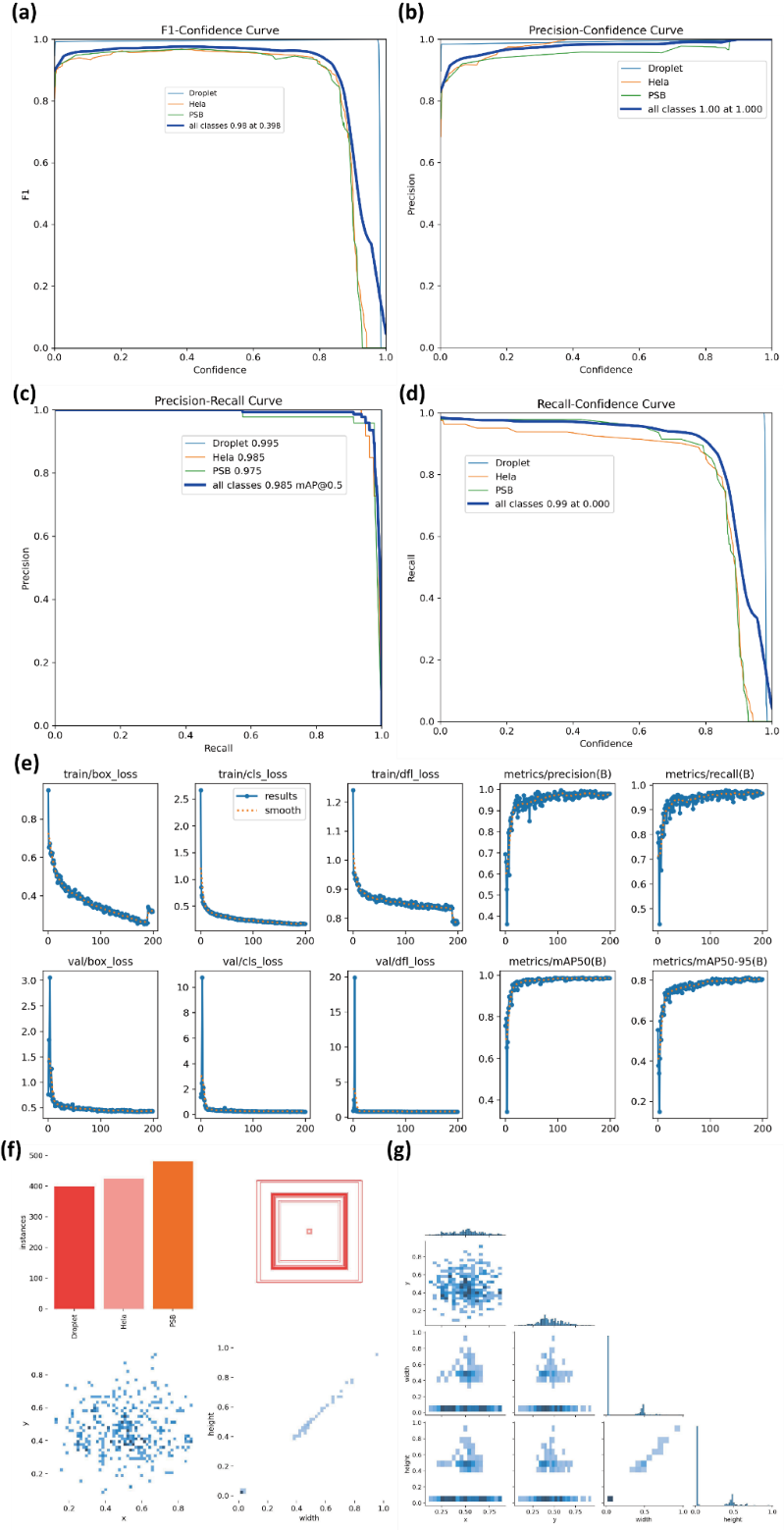


**Figure S2.** Training results of the object detection model on the HeLa-PSB dataset. (a)-(d) F1-Confidence curve, Precision-Confidence curve, Precision-Recall curve and Recall-Confidence curve of the model, respectively. (e) The changes of each metric in the training process. (f) The number, coordinates, and distribution of height and width of each type of instances in the training set. (g) Correlograms of x, y, height and width of each type of instances in the training set.


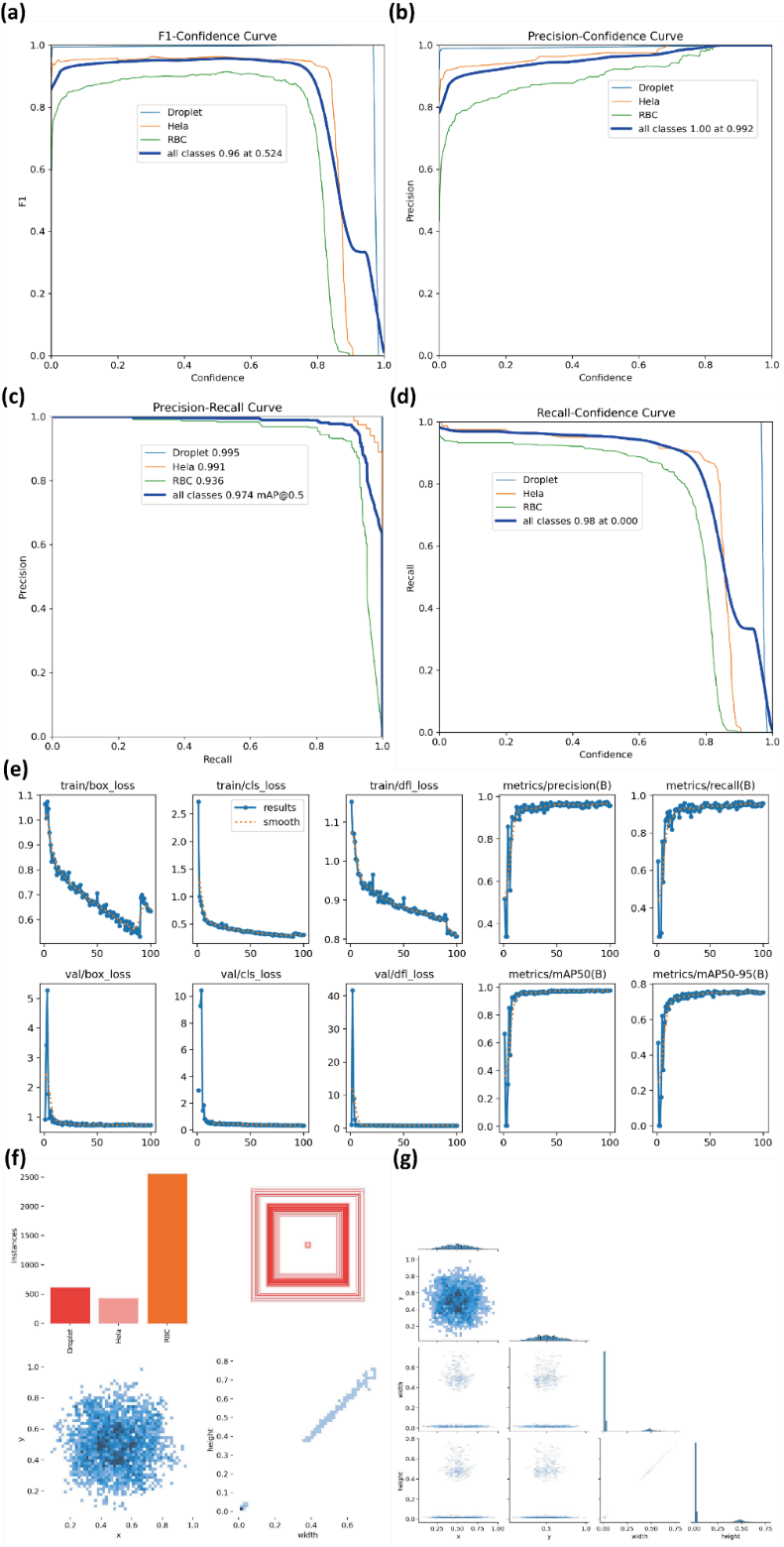


**Figure S3.** Training results of the object detection model on the HeLa-RBC dataset. (a)-(d) F1-Confidence curve, Precision-Confidence curve, Precision-Recall curve and Recall-Confidence curve of the model, respectively. (e) The changes of each metric in the training process. (f) The number, coordinates, and distribution of height and width of each type of instances in the training set. (g) Correlograms of x, y, height and width of each type of instances in the training set.


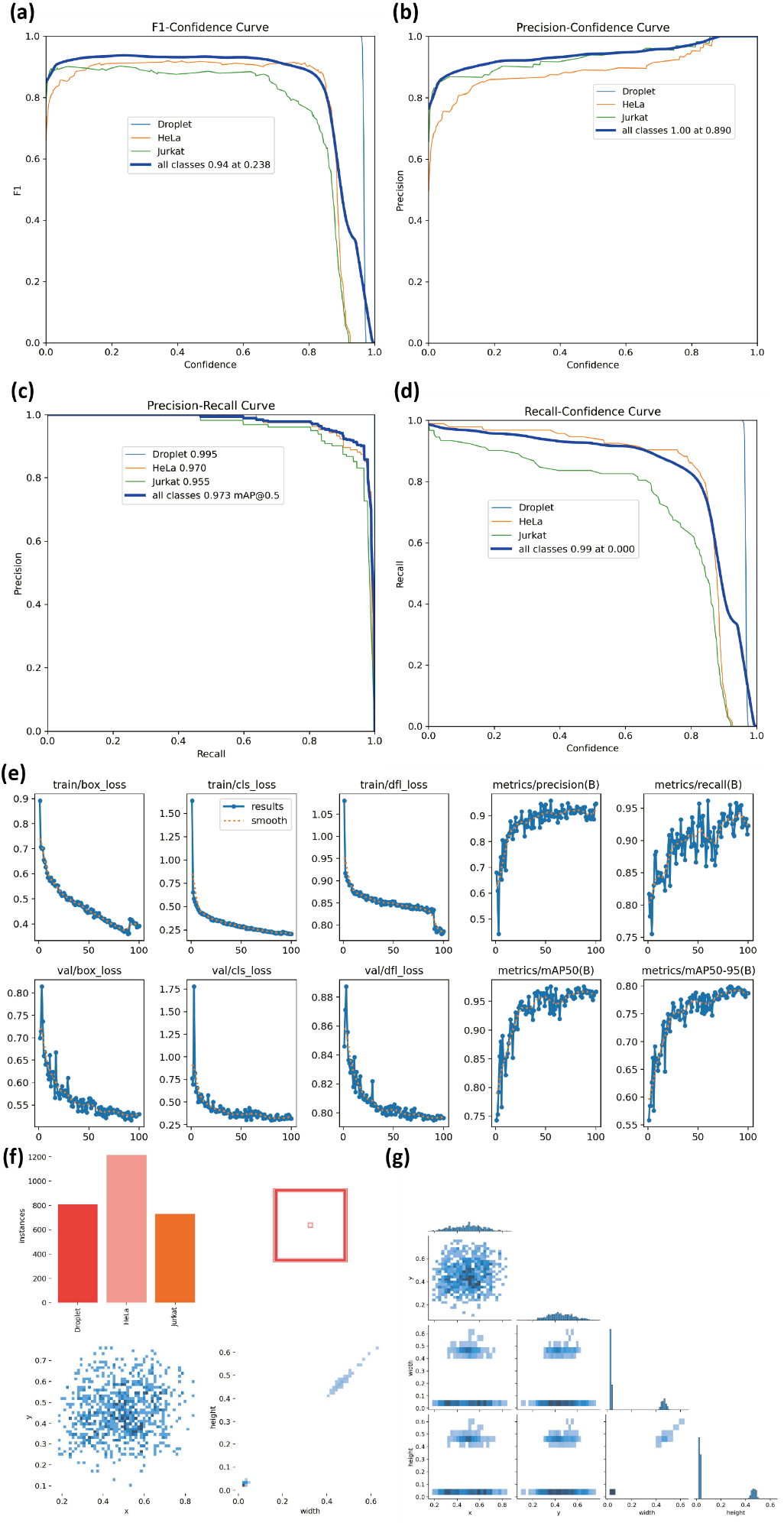


**Figure S4.** Training of the object detection model on the HeLa-Jurkat dataset. (a)-(d) F1-Confidence curve, Precision-Confidence curve, Precision-Recall curve and Recall-Confidence curve of the model, respectively. (e) The trends of each metric in the training process. (f) The number, coordinates, and distribution of height and width of each type of instances in the training set. (g) Correlograms of x, y, height and width of each type of instances in the training set.


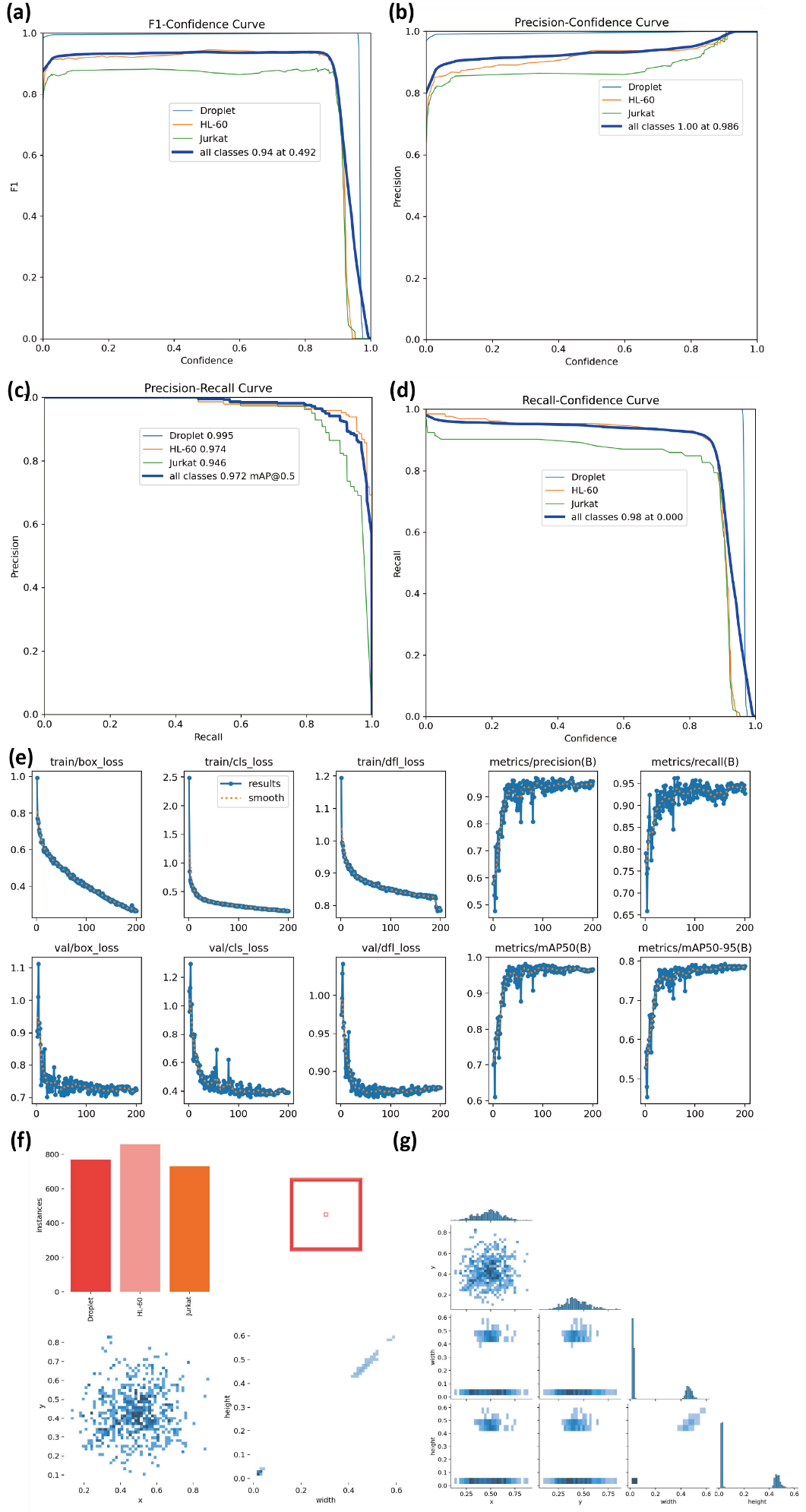


**Figure S5.** Training of the object detection model on the HL-60-Jurkat dataset. (a)-(d) F1-Confidence curve, Precision-Confidence curve, Precision-Recall curve and Recall-Confidence curve of the model, respectively. (e) The trends of each metric in the training process. (f) The number, coordinates, and distribution of height and width of each type of instances in the training set. (g) Correlograms of x, y, height and width of each type of instances in the training set.

| **Ground Truth** | **Prediction** |
| --- | --- |
| 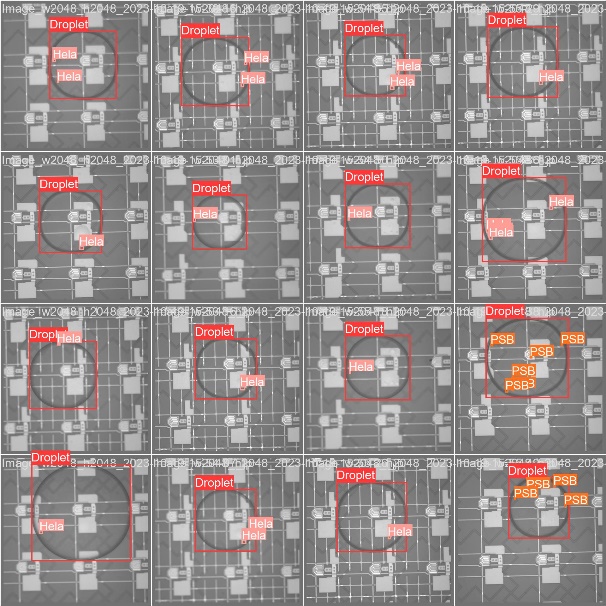 | 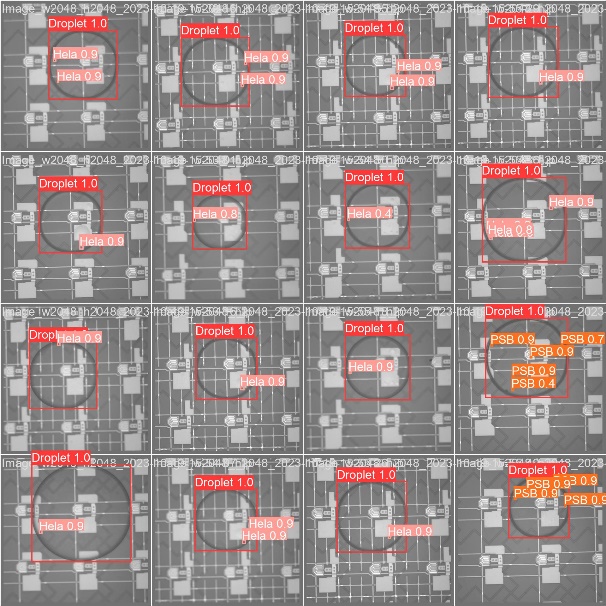 |
| 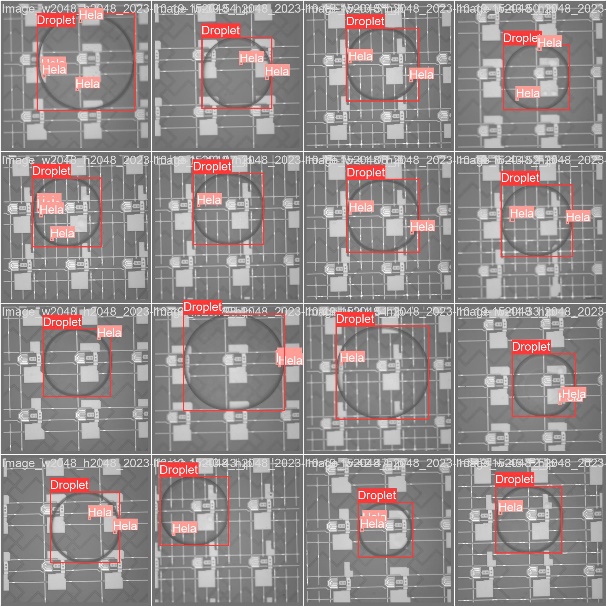 | 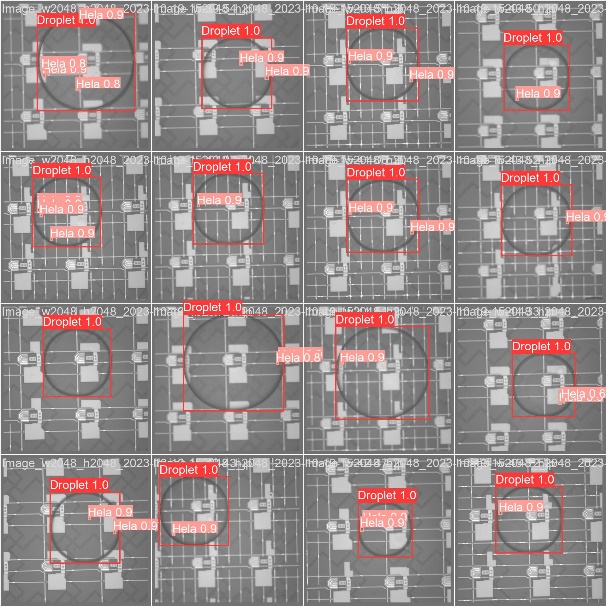 |

**Figure S6.** Ground truths and predictions of the validation set of the HeLa-PSB dataset. The location and type of cells in the droplets shown in the model prediction results on the right side correspond well with the real results on the left side, demonstrating the precision of the model identification.

| **Ground Truth** | **Prediction** |
| --- | --- |
| 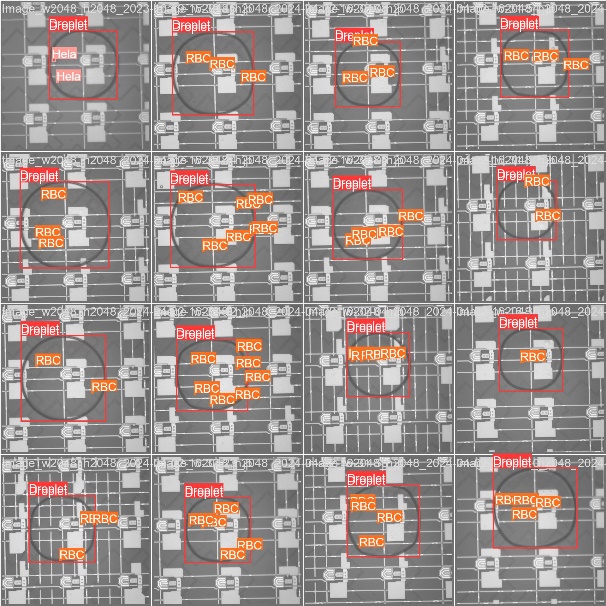 | 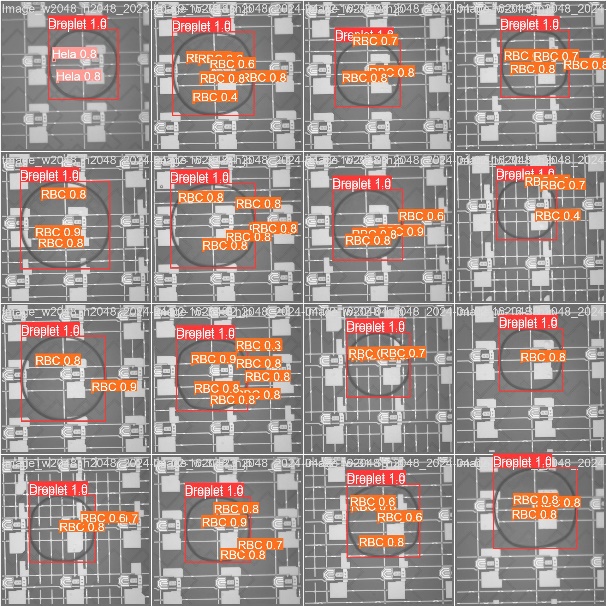 |
| 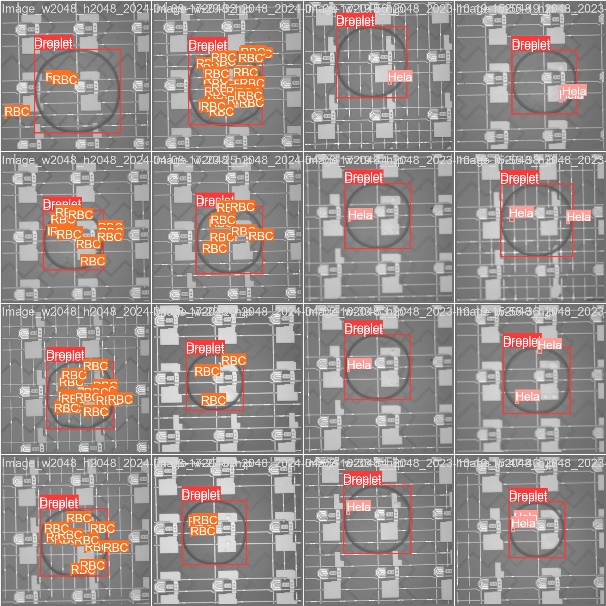 | 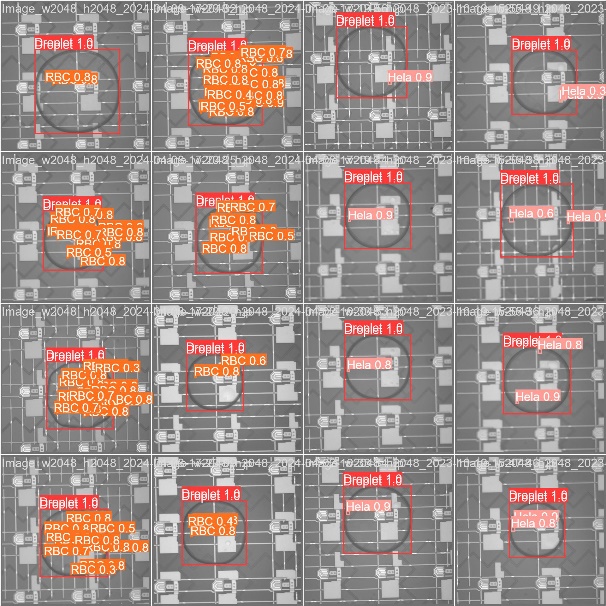 |

**Figure S7.** Ground truths and predictions of the validation set of the HeLa-RBC dataset. The location and type of cells in the droplets shown in the model prediction results on the right side correspond well with the real results on the left side, demonstrating the precision of the model identification.

| **Ground Truth** | **Prediction** |
| --- | --- |
| 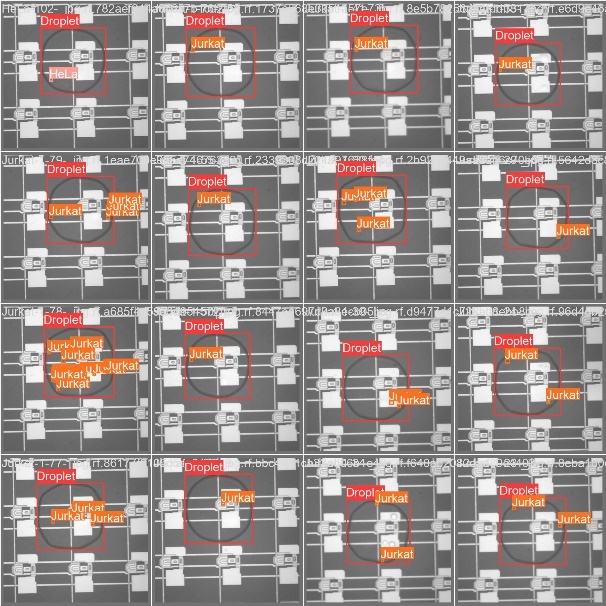 | 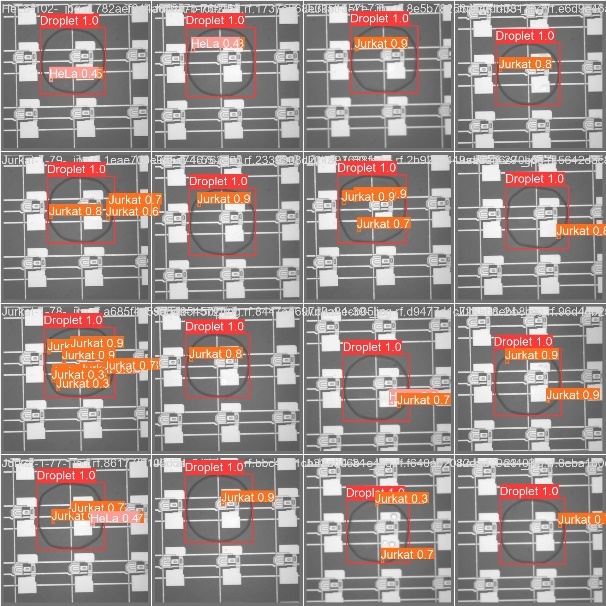 |
| 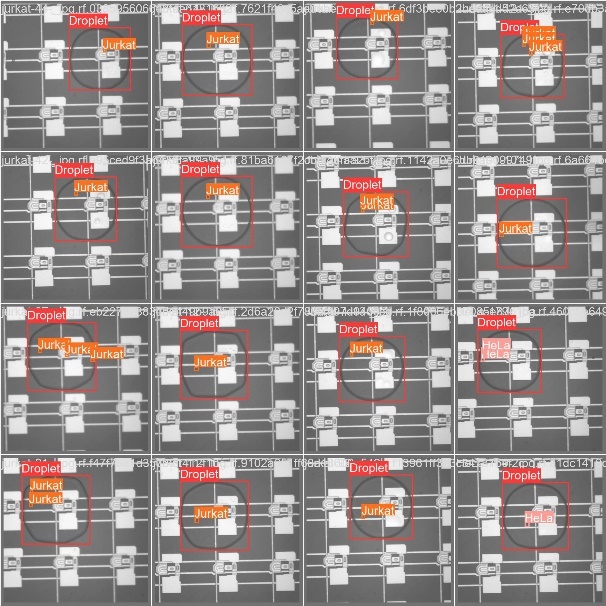 | 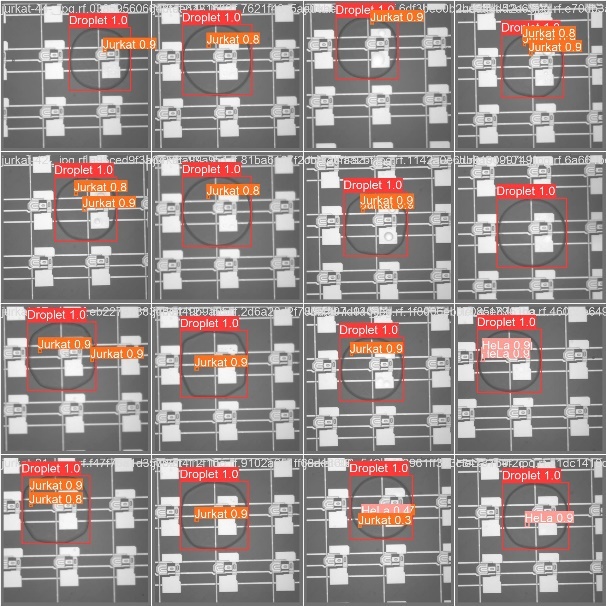 |

**Figure S8.** Ground truths and predictions of the validation set of the HeLa-Jurkat dataset. The location and type of cells in the droplets shown in the model prediction results on the right side correspond well with the real results on the left side, demonstrating the precision of the model identification.

| **Ground Truth** | **Prediction** |
| --- | --- |
| 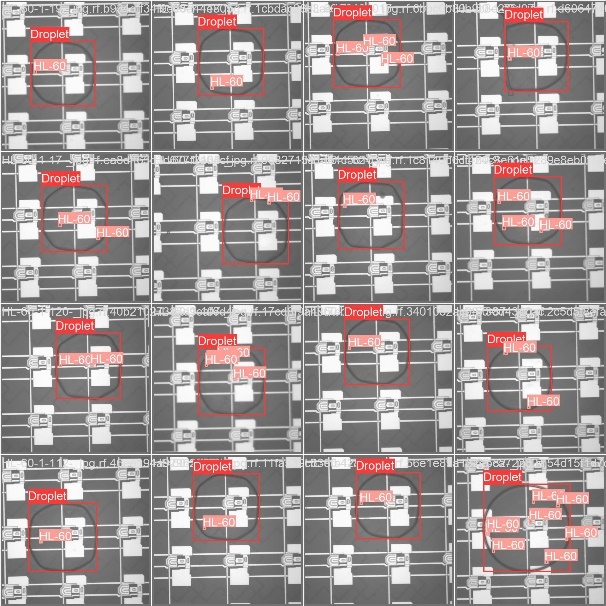 | 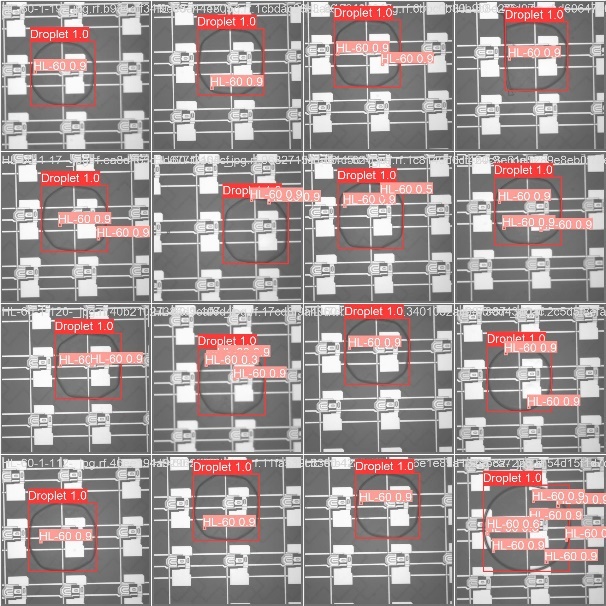 |
| 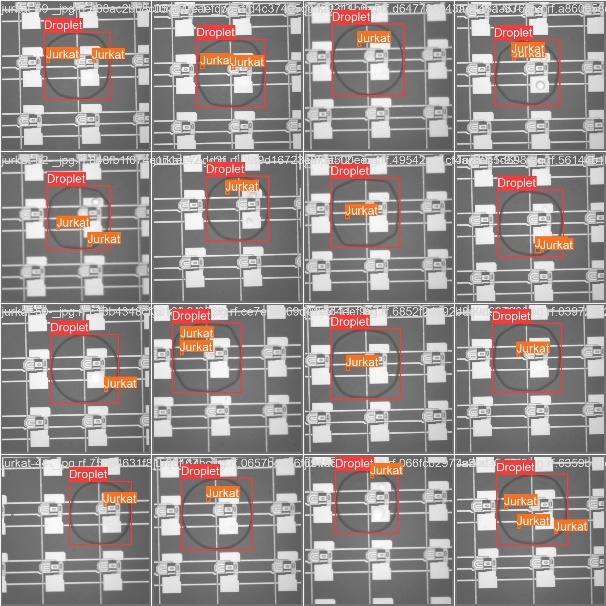 | 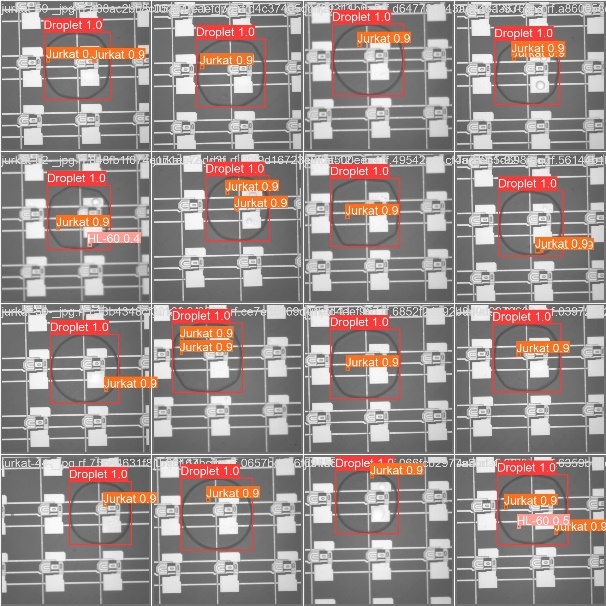 |

**Figure S9.** Ground truths and predictions of the validation set of the HL-60-Jurkat dataset. The location and type of cells in the droplets shown in the model prediction results on the right side correspond well with the real results on the left side, demonstrating the precision of the model identification.


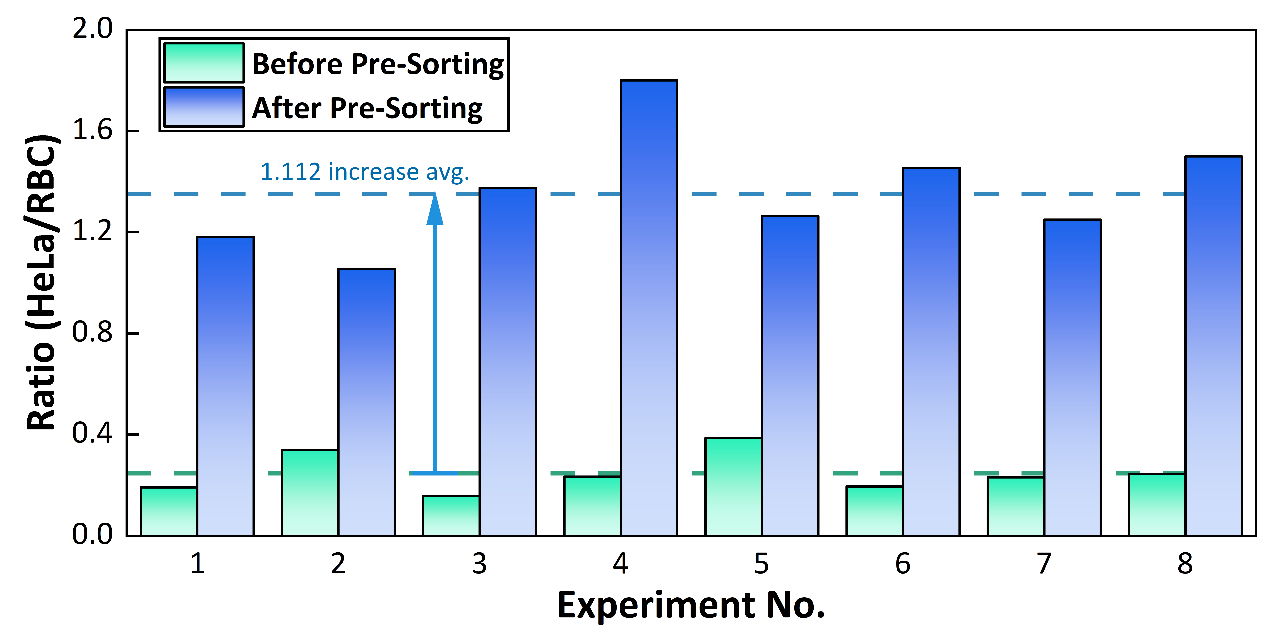


**Figure S10.** Comparison of the concentration ratio of target cells before and after pre-sorting in 8 independent rare cell sorting experiments.

**Supplementary Movies**

**Movie S1:** Experimental video showing the process of cell sorting with programmable droplets on AM-DMF chip (**Fig. 1b**).

**Movie S2:** Experimental video showing the process of on-chip cell lysis by merging and mixing two droplets (**Fig. 5**).
